# Modifications of auditory feedback and its effects on the voice of adult subjects: a scoping review

**DOI:** 10.1590/2317-1782/20232022202en

**Published:** 2023-12-22

**Authors:** Moisés do Carmo Alves, Patrícia Cotta Mancini, Leticia Caldas Teixeira

**Affiliations:** 1 Programa de Pós-graduação em Ciências Fonoaudiológicas, Departamento de Fonoaudiologia, Faculdade de Medicina, Universidade Federal de Minas Gerais – UFMG - Belo Horizonte (MG), Brasil.

**Keywords:** Auditory Feedback, Voice Training, Adult, Auditory Perception, Feedback, Study Review

## Abstract

**Introduction:**

The auditory perception of voice and its production involve auditory feedback, kinesthetic cues and the feedforward system that produce different effects for the voice. The Lombard, Sidetone and Pitch-Shift-Reflex effects are the most studied. The mapping of scientific experiments on changes in auditory feedback for voice motor control makes it possible to examine the existing literature on the phenomenon and may contribute to voice training or therapies.

**Purpose:**

To map experiments and research results with manipulation of auditory feedback for voice motor control in adults.

**Method:**

Scope review following the Checklist Preferred Reporting Items for Systematic reviews and Meta-Analyses extension (PRISMA-ScR) to answer the question: “What are the investigation methods and main research findings on the manipulation of auditory feedback in voice self-monitoring of adults?”. The search protocol was based on the Population, Concept, and Context (PCC) mnemonic strategy, in which the population is adult individuals, the concept is the manipulation of auditory feedback and the context is on motor voice control. Articles were searched in the databases: BVS/Virtual Health Library, MEDLINE/Medical Literature Analysis and Retrieval System online, COCHRANE, CINAHL/Cumulative Index to Nursing and Allied Health Literature, SCOPUS and WEB OF SCIENCE.

**Results:**

60 articles were found, 19 on the Lombard Effect, 25 on the Pitch-shift-reflex effect, 12 on the Sidetone effect and four on the Sidetone/Lombard effect. The studies are in agreement that the insertion of a noise that masks the auditory feedback causes an increase in the individual's speech intensity and that the amplification of the auditory feedback promotes the reduction of the sound pressure level in the voice production. A reflex response to the change in pitch is observed in the auditory feedback, however, with particular characteristics in each study.

**Conclusion:**

The material and method of the experiments are different, there are no standardizations in the tasks, the samples are varied and often reduced. The methodological diversity makes it difficult to generalize the results. The main findings of research on auditory feedback on voice motor control confirm that in the suppression of auditory feedback, the individual tends to increase the intensity of the voice. In auditory feedback amplification, the individual decreases the intensity and has greater control over the fundamental frequency, and in frequency manipulations, the individual tends to correct the manipulation. The few studies with dysphonic individuals show that they behave differently from non-dysphonic individuals.

## INTRODUCTION

Hearing is greatly important to voice production and self-monitoring, and its influence on voice production has been addressed in the scientific literature^(1-7)^. Voice production and its monitoring involve three mechanisms: auditory feedback, kinesthetic cues (or somatosensory feedback), and the feedforward system^(7)^.

Auditory feedback is the hearing perception of one’s own voice in real time, enabling the person to monitor its intensity, frequency, and quality^(5,8-10)^. Somatosensory feedback is the perception of adaptations and the motor adjustments of structures involved in the phonating process^(11)^.

Auditory and somatosensory feedback help produce internal references for speech motor planning and update these adjustments for the feedforward system^(9)^ – which is theoretically described as a cortical system located in the left brain hemisphere. It is responsible for mapping the articulation movements of the lips, mandible, tongue, and larynx and stores these speech-motor adjustments based on motor, somatosensory, and auditory references^(8)^. The feedforward system uses these previously acquired internal references to control the voice^(7)^.

Different types of auditory feedback manipulations produce distinct effects on the person’s voice – of which the most studied ones are the Lombard effect, the sidetone or amplification effect^(12-14)^, and the pitch-shift effect^(15-18)^.

The Lombard effect occurs when the intensity of voice production increases by inserting an intense noise, which masks the auditory feedback. The voice’s sound pressure is increased unconsciously and instantaneously, and when the noise is removed, vocally healthy people tend to return to the speech intensity level they were using before the noise was inserted^(2,18)^.

The sidetone effect is the amplification of the sound feedback, increasing the person’s perception of their own voice^(12)^. In response to this manipulation, vocally healthy people reduce their voice’s sound pressure level^(12)^. Hence, this effect reduces the sound pressure of the voices of patients with hyperfunctional dysphonia and creates or increases the subject’s auditory perception regarding parameters and changes in their voice^(8)^, making it possible to monitor its fundamental frequency, quality, and intensity^(8,12-14)^.

The pitch-shift effect occurs when a person with no vocal changes is auditorily exposed to changes in their own voice’s frequency. This manipulation causes a reflex correction, which is known to change the frequency most commonly in the opposite direction of the manipulation. Another less often possibility is to change it in the same direction of the manipulation^(10,15-17)^.

It is important to understand how vocal changes produced by different auditory feedback manipulations can be used in voice therapy and training. Little is known about experiments regarding the time of exposure to feedback, the level of amplification or masking noise, and the effectiveness of these manipulations for the voice of individuals with and without vocal complaints, whether they are occupational voice users or not. It is believed that mapping the literature on the topic through a scoping review will help examine the current scientific literature on the phenomenon and verify its gaps, envisioning future possibilities for further studies.

Given the above, the following question was raised: “What are the investigation methods and main findings in research on auditory feedback manipulation in adults’ self-monitoring of voice?”.

## METHOD

This scoping review followed the detailed checklist of the Preferred Reporting Items for Systematic Reviews and Meta-Analyses – Extension for Scoping Reviews (PRISMA-ScR) in the Joanna Briggs Institute Reviewers’ Manual^(19)^. This checklist has 22 items that guide the writing of the scoping review report. The review was conducted between November 2021 and November 2022. The review protocol was registered in the Open Science Framework (OSF) on November 29, 2021, under DOI:10.17605/OSF.IO/CYM9N.

It used Arksey and O’Malley’s methodological framework^(20)^ with Levac et al.^(21)^ and Peters et al.^(22)^ recommendations: 1) identifying the research question and objective; 2) identifying relevant studies; 3) selecting studies; 4) mapping data; 5) selecting evidence; 6) presenting results.

Two researchers selected, included, and extracted data independently regarding study eligibility for selection and inclusion. Inclusion and exclusion criteria were defined as follows, according to PRISMA-ScR:

Inclusion criteria: articles with the term “auditory feedback” in their titles and abstracts; articles addressing the topic implicit in their abstracts, related to speech motor control in adults, whether or not occupational voice users; addressing voice treatment or training; articles written in Portuguese, English, or Spanish, with no restriction on the year. These criteria were established to screen all literature available on the topic.

The exclusion criteria were articles using auditory feedback in contexts other than the subjects’ own voice sound amplification (sidetone effect), auditory feedback suppression (Lombard effect), or voice frequency manipulation (pitch-shift effect); articles using feedback with external target sounds; and articles whose populations had neurological conditions. Articles whose topic was unclear in their titles or abstracts were assessed in full texts. In cases of divergence, the reviewers analyzed the articles together to define by consensus whether to keep or remove them.

### Search strategy – Research question and search criteria

The review question was developed based on the PCC mnemonic strategy, corresponding to Population, Concept, and Context^(19,22)^. In this research, the population referred to adults; concept, to auditory feedback manipulation; and context, to speech motor control.

The sources of evidence were scientific studies published up until November 2022, addressing the use of auditory feedback, including every type of evidence. The articles were searched in the following databases: VHL (Virtual Health Library), MEDLINE (Medical Literature Analysis and Retrieval System Online), Cochrane, CINAHL (Cumulative Index to Nursing and Allied Health Literature), Scopus, and Web of Science. The DeCS/MeSH descriptors used for the concept of “auditory feedback” were Auditory Perception, Audio Feedback; and the keywords were Auditory Feedback, Auditory, External Auditory, Internal Auditory, Kinesthetic, Portable Amplification, Sound Amplification, Auditory Masking, Auditory Self-Monitoring, Sidetone, Pitch-Shift Auditory, Frequency, Shifted, Lombard Effect. As for the concept of “speech motor control”, the descriptors were Voice Quality, Voice Training, Voice; and the keywords were Voice Motor Control, Voice Control, Pitch Control, Control of Voice Intensity.

**Figure 1:**
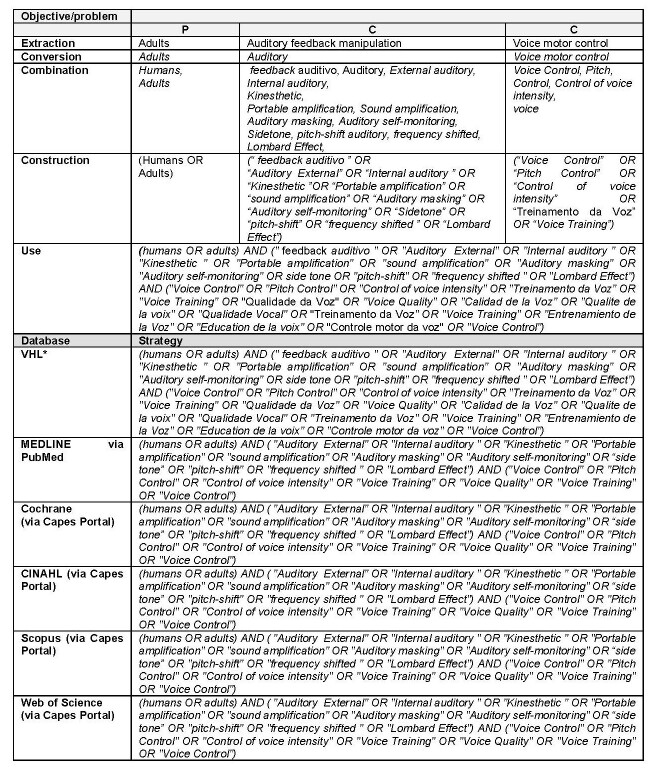
Development of the search strategy

Figure 1 shows the PCC search strategy developed by the lead researcher and reviewed by the second researcher. After the search, the references in the retrieved articles were also manually searched to complement the data.

### Mapping data

After selecting the studies, they were exported to the Rayyan platform – Intelligent Systematic Review. They were blindly selected by each researcher, and the inclusion of conflicting studies was defined by consensus among researchers. After this selection, the form was discussed and updated according to the data they considered relevant. The studies were grouped per type of auditory feedback manipulation, observing their designs, populations, tasks used in experiments, and the dependent variables used to measure the results. Duplicates were removed, and data were extracted and organized in spreadsheets, according to the type of feedback, with the study information relevant to this review: author, country, year, objectives, method, main results, and conclusion.

### Selecting evidence

After categorizing the studies, their results were summarized in two charts to make information comparison easier.

## RESULTS

**Figure 2:**
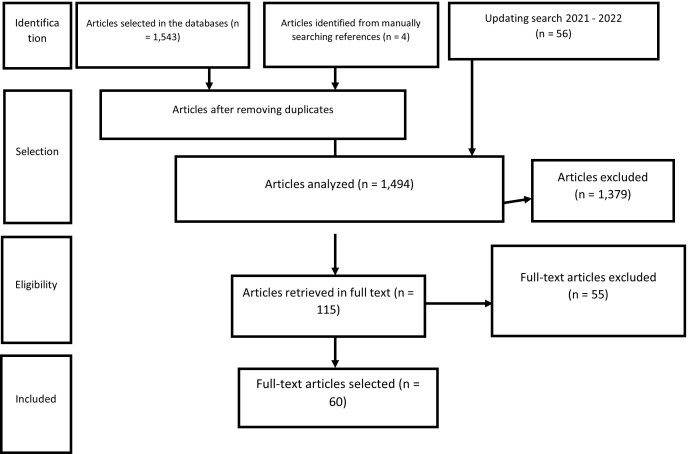
Flowchart of the search in the literature and inclusion of articles

The synthesis of the results of the identification, selection, eligibility, and inclusion phases is described in the organogram shown in Figure 2.

The search found 19 studies (corresponding to 31.65%) that used the Lombard effect, all of them cross-sectional. Most samples comprised both male and female participants (n = 15), non-occupational voice users (n = 16), and without vocal complaints (n = 15). Only one study included individuals with dysphonia, and five studies approached singers. The tasks mainly used vowels, followed by singing and then reading. The inserted masking noise ranged from 50 to 105 dB, as there is no consensus or standardization for noise measure. Acoustic measures were the most studied outcomes, especially F0. Results agree that inserting noise to mask auditory feedback increases the person’s speech intensity. Articles that researched adult singers agree that auditory feedback, even in different proportions according to the training level, contributes to tuning precision in singing. Individuals with voice problems seemingly behave differently, having difficulties in returning to the usual adjustment after being exposed to the masking noise.

Also, 12 articles (20% of those found) researched the sidetone (amplification) effect in auditory feedback (Chart 1) – 11 are cross-sectional experimental studies, and the other one is a randomized clinical trial. Most studies focused on the effects of amplification on the human voice. Most samples had both male and female (n = 6) occupational voice users (n = 7), most of them teachers/professors (n = 6). Three studies approached dysphonic subjects, and one had participants with and without vocal complaints. The most used tasks were spontaneous speech (n = 8), followed by text reading (n = 3). Only one study used singing samples. The intensity of voice amplification was controlled in some studies, while other ones did not control it. Acoustic analysis was the most studied outcome, focusing on changes in sound pressure and the subjects’ self-perception. The studies concordantly found decreased sound pressure levels when the auditory feedback is amplified and positive voice production effects when it was amplified in the study samples.

**Chart 1:** Studies on manipulations of auditory feedback intensity (Lombard Effect, Sidetone Effect, and Sidetone/Lombard Effect) according to authorship, publication year, study country, objective, method, and conclusions

**Lombard Effect**					
**Author, country, year**	**Objectives**	**Sample**	**Task**	**Variables**	**Main findings and conclusion**
1.Alghamdi et al., England, 2018	To characterize the acoustic, phonetic, and articulatory modifications of speech in the Lombard Effect.	54 speakers, both genders.	Read 100 randomized sentences with noise exposure of 80 dB.	f0, mean volume, spectral energy, mean vowel duration	There were acoustic and articulatory changes for all participants. In the largest increase in the estimated duration of the vowel, there was a significant reduction in the frequency of the second formant.
2.Castro et al., Chile, 2018.	To compare the aerodynamic, biomechanical and neurophysiological acoustic parameters of healthy individuals and individuals with dysphonia when exposed to the Lombard effect.	10 individuals, both genders, healthy and with muscle tension dysphonia.	Pronounce a series of vowels and syllables, displayed on a screen, with and without noise of 80 dB and after using noise.	Videolaryngoscopy, aerodynamic and acoustic measurements.	Subjects with muscle tension dysphonia may be more sensitive to the Lombard effect and have greater difficulty returning to their usual settings. It is believed that these patients have an interrupted auditory-motor control integration during speech production.
3.Fernandes et al., Brazil, 2018.	Evaluate the influence of auditory feedback on voice intensity and frequency in individuals without vocal complaints	40 women without vocal complaints.	Producing the vowel /a/, saying the days of the week and singing before, during and after exposure to 80 dB white noise in headphones.	Voice intensity and frequency, before, during and after exposure to noise.	The condition of exposure to noise causes an increase in voice intensity and the interruption of exposure to noise causes a decrease in vocal intensity in women without complaints.
4. Lijima et al., Japan, 2016.	To investigate the effects of auditory feedback masking in a singing task.	6 men.	Sing a firm /a/ vowel in tones: C3, G3 and C4 for 5 seconds under 85 dB pink noise, 85 dB pink noise with a 2 kHz how pass filter and no masking.	F0, sound pressure level and formants 1 and 2 in each condition.	Sound pressure level and formant 1 and 2 frequencies increased under noise in both experiments. The sound pressure level and formant 1 and 2 frequencies decreased when the 2 kHz high pass filter was used.
5. Kleber et al., Canada, 2016.	To test how auditory feedback masking affects pitch accuracy and corresponding brain activity in trained and untrained singers.	22 singers, (4 M e 19 F), divided into trained and untrained.	Hear and sing tones (Between C# 3 to D5 for women and F2 and B3 for men), with and without masking inserted through headphones.	Magnetic resonance imaging during exposure to noise.	Pitch accuracy matching was unaffected by masking in trained singers, but decreased in non-singers. The right insula was up-regulated during masking in singers, but down-regulated in non-singers. Functional connectivity with inferior parietal, frontal, and sensorimotor areas relevant to voice increased in singers but decreased in non-singers.
6. Yiu and Yip, China, 2015.	Investigate the effects of environmental noise on vocal intensity and f0 using an accelerometer.	24 young adults (2 groups of 12 F and 12 M).	Read text, from 3 to 5 minutes, under the conditions: (1) quiet room (35.5 dB); (2) room with moderate level of ambient noise (54.5 dB); (3) a room with high ambient noise (67.5 dBA).	Variations in F0, perception of effort, sound pressure level, and vocal dose measurements.	Both groups showed increases in vocal intensity, F0, and perception of vocal effort in the high-noise environment compared to the other two conditions. The results support that conversation noise levels should be maintained <50-55 dB to preserve speech intelligibility.
7. Erdemir and Rieser, USA, 2012.	To investigate the effects of noise exposure on the control of singing abilities in trained and untrained singers.	42 individuals; both sexes. Singer group, instrumentalist group, and non-musician group.	To sing the same song under conditions with and without masking (conversation and music sound) played through headphones at 95dB. Participants were instructed to try to maintain vocal intensity at 80dB based on visual feedback.	Acoustic analysis, F0 variations.	Auditory feedback is an important factor in maintaining pitch and timing accuracy even after years of musical training. Singers relied less on auditory feedback. Instrumentalists and non-musicians were impaired by the absence of auditory feedback.
8.Li and Jeng, China, 2011.	To examine voice pitch maintenance during modifications in auditory feedback, observing the effects of signal-to-noise ratio at various stimulus intensities.	12 adults (5 M e 7 F)	To produce the vowel /i/ under six conditions of signal-to-noise ratio (12, 6, 0, 6, and 12 dB) at three different intensities of auditory masking through headphones (70, 55, and 40 dB) during phonation.	Electroencephalography, F0 variations.	There is a tolerance for pitch control in relation to noise. There is a minimum signal-to-noise ratio to assess pitch processing.
9. Caldeira et al., Brazil, 2011.	To verify and compare the occurrence of vocal modifications in reporters and non-reporters in the presence of masking noise.	46 subjects, both sexes, allocated as follows: 23 reporters and 23 non-reporters.	To read a passage from a newspaper article (36 words) under the following conditions: without noise, with 50 dB noise, and with 90 dB noise. The noises were introduced through headphones.	Auditory perceptual evaluation and acoustic analysis.	With 50 dB of masking, there was a greater increase in the pitch (82.6%), loudness (91.3%), and tension (82.6%) parameters in the control group compared to the reporters' group. The same occurred with 90 dB noise for the pitch (95.7%), loudness (100%), and tension (91.3%) parameters. Reporters demonstrated partial inhibition of the negative impact of noise situations.
10. Grillo et al., USA, 2010.	Explore the effects of auditory masking on laryngeal resistance when individuals produced breathy, normal, and tense voices of trained women.	18 vocally trained women.	Produce breathy, normal, and tense voices at 7 fundamental frequencies (220 Hz, 277 Hz, 349 Hz, 440 Hz, 554 Hz, 698 Hz, and 880 Hz) during a repeated /pi/ utterance under normal auditory feedback and masked with 104 dB noise.	Mean and standard deviation of laryngeal resistance, aerodynamic measurements.	The values of laryngeal resistance for breathy and normal voice remained constant in both feedback conditions, while for tense voice, they increased in the masked feedback. Tense voice may be more susceptible to the influence of auditory feedback because it is less stable than the other tested patterns.
11. Lindstrom et al., Sweden, 2009.	To investigate the correlations between noise level and fundamental frequency (F0) in a population of preschool teachers at their workplace.	13 preschool teachers.	Usual teaching activity of 3 to 4 hours of class while measuring vocal production intensity (through a microphone attached near the mouth) and the noise level of the environment (measured by a decibel meter).	Vocal dose, sound pressure level of the voice, and local noise level.	Vocal behavior in relation to noise exposure is highly individual based on the analyzed parameters. The reduction in noise level did not necessarily correspond to a reduction in sound pressure level emitted by the participant.
12 Larson et al., USA, 2008.	Test the vocal modifications in fundamental frequency (F0) and amplitude control during simultaneous perturbations of voice pitch and intensity auditory feedback.	24 subjects tested (2 males and 22 females).	Sustain a vowel /u/ under the following conditions: 1. Change the frequency by 0.5 semitones up or down; 2. Change the intensity by 10 dB above the produced signal; 3. Change the intensity and frequency according to the previously presented patterns.	Modifications of intensity and F0.	The subjects responded in the opposite direction to the frequency or intensity displacement stimuli. Depending on the direction of the stimulus, both responses can change either in the same direction or in the opposite direction to each other.
13. Lee et al., USA, 2007.	To investigate the relationship between auditory function and F0 using binaural masking with noise during sustained vowel vocalizations.	8 healthy individuals (4 males and 4 females).	​​ Produce sustained vowel /a/ at intensities of 65 to 75 dBA and 90 to 100 dBA with and without the presence of 85 dB noise inserted through headphones.	Modifications of F0.	There was an increase in the frequency range of <3 Hz during the noise insertion. A negative feedback control on F0 is suggested regarding F0 modulations smaller than 3 Hz. The auditory system helps control the stability of F0 during sustained vowel production.
14. Ferrand, EUA, 2005.	To investigate phonatory stability by measuring changes in intensity, F0, jitter, and NHR in different noise conditions.	22 women without complaints.	Three sustained emissions of the vowel /a/ for each condition: 1. Noise level (0-dB ML); 2. 50 dB noise inserted through headphones; 3. 80 dB noise inserted through headphones.	Acoustic measurements of frequency and intensity.	There was an increase in vocal production intensity in both noise conditions. There was also an increase in fundamental frequency (F0), although it was less robust.
15. Deliyski et al., USA, 2005.	To investigate the influence of noise on the accuracy, reliability, and validity of acoustic measures of voice quality for gender, age, intersubject and intrasubject variability.	20 participants of both genders.	Produce sustained vowel /a/ for 10 seconds at 88 dB, inserted through headphones, during the presence and absence of noise at levels 42 dB above, 30 dB above, and 30 dB below the production intensity.	Acoustic measurements.	The results suggest that the recommended, acceptable, and unacceptable levels of noise in the acoustic environment are above 42 dB, above 30 dB, and below 30 dB signal-to-noise ratio, respectively.
16. Mürbe et al., Germany, 2003.	To evaluate the effect of training on singing control in legato and staccato tasks, at slow and fast tempos, in students with 3 years of musical education.	22 trained singers, both genders.	Sing the vowel /a/ in an ascending scale and a descending triad pattern covering your entire pitch range, with and without 105dB masking, inserted through headphones, in legato and staccato, and at a slow and fast tempo.	F0 and comparison between intervals and pitch accuracy.	The masking compromised the accuracy of pitch, for both staccato and legato, and for fast performance compared to slow performance. Kinesthetic feedback contributes to pitch accuracy in trained singers.
17. Mürbe et al., Germany, 2002.	To estimate the importance of auditory and kinesthetic feedback for voice pitch control in 28 beginner professional solo singing students.	28 singers, both genders (17 females and 11 males).	Sing the vowel /a/ in an ascending scale and a descending triad pattern covering your entire pitch range, with and without 105dB masking inserted through headphones, in legato and staccato, and at a slow and fast tempo.	Measurement of F0 accuracy using software.	The masking compromised pitch accuracy by 14% in all subjects in the conditions of fast tempo, staccato, and legato. Auditory feedback contributes to the pitch control of singers.
18. Tonkinson, USA, 1994.	To compare the level of vocal intensity response of adult singers with different training durations before and after verbal instructions to resist the Lombard Effect while singing with a pre recorded tape of a choir singing.	27 individuals of both genders.	Sing and ignore, through verbal commands, the Lombard Effect produced by inserting audio, through headphones, at intensities of 80 to 100 dB, containing recordings of other people singing the same musical excerpt.	Sound pressure level.	Both groups were able to resist the Lombard Effect based on simple commands given by the evaluator.
19. Herbert et al., USA, 1988.	To test the resistance to the Lombard Effect when individuals are instructed and trained with visual feedback to suppress it.	24 students, both genders, assigned to: G1 (constant vocal intensity for 2 minutes alternating periods of silence and noise with visual feedback to resist the Lombard Effect), G2 (same instructions as G1, but without feedback), G3 (no instruction or visual feedback).	Speak spontaneously during the session for 20 minutes while receiving white noise of 90 dB through headphones.	Variations in speech intensity.	Individuals who had visual support were able to inhibit the Lombard response, and the inhibition remained after the visual feedback was removed. The Lombard response is largely automatic and involuntary.
**Sidetone Effect**					
**Author, country, year**	**Objectives**	**Sample**	**Task**	**Variables**	**Main findings and conclusion**
1. Nudelman et al., USA, 2021.	To examine the effects of auditory feedback amplification through bone conduction on acoustic vocal parameters and subjective self-assessment of vocal effort in patients with vocal disorders.	47 dysphonic individuals (14 males and 13 females).	Perform reading of texts under three conditions: auditory feedback amplification at 54 dB HL and 58 dB HL, and without amplification. Amplification was done through a microphone and headphones.	Self-perceived and recorded vocal effort on a visual analog scale (EVA), vocal sound pressure level adaptation level.	There is a consistent positive adaptation in sound pressure level for vocal hyperfunction, glottic insufficiency, and laryngeal pathologies when subjected to an amplification task with higher intensity (58dB).
2. Tomassi et al., USA, 2021.	To determine the therapeutic potential of amplification effects on vocal function during audiovisual telecommunications.	18 participants (8 males and 10 females).	Conversation task under three conditions: without amplification, with low sidetone amplification, and with high sidetone amplification, for 10 minutes.	Vocal intensity, vocal quality, and self-perceived effort.	There were decreases in vocal intensity during the auditory feedback amplification condition, and participants perceived less vocal effort during the amplification. Results indicated a possible improvement in vocal quality.
3. Assad et al., Brazil, 2017.	To determine if voice amplification influences vocal dose in female teachers with dysphonia.	15 female teachers with functional dysphonia.	Two assessment moments. 1st Moment: Teaching with a portable electronic sound amplification system for 92 minutes; 2nd Moment: Teaching without a sound amplification system for 92 minutes.	Intensity, fundamental frequency, phonation percentage, cyclic dose, and distance dose.	The use of vocal amplification in teachers results in a reduction in F0 (fundamental frequency) and voice intensity. The cyclic dose and distance dose show that amplification allows the teacher to maintain the same phonation time but decreases the number of vocal fold oscillations (cyclic dose) and the total distance traveled by the vocal fold tissue during phonation (distance dose), reducing vocal fold exposure to vocal trauma.
4. Gaskill et al., USA, 2011.	To determine the effect of a portable voice amplifier on vocal dose in teachers with and without vocal complaints.	2 teachers, one with vocal complaints and one without vocal complaints.	Teach for one week with and one week without the use of a portable voice amplification system, while using a vocal dosimeter throughout the experiment. Each week lasted for five days, with one session per day.	Cyclic, dose distance and intensity.	The use of the amplifier was effective in reducing vocal load due to the decrease in speech intensity. Amplification reduces the distance dose and appears to decrease the cyclic dose.
5. Nsdottira et al., Iceland, 2003.	To investigate changes in teachers' voice quality during two situations: a regular workday under normal conditions and another with sound amplification.	5 teachers (3 females and 2 males).	Teach during the most challenging workday under both normal conditions and amplified conditions using a microphone system and loudspeaker calibrated to a maximum signal of 80dB. Recordings were conducted during the first and last class of the teacher's shift, each lasting 40 minutes.	F0 (fundamental frequency), sound pressure level, and questionnaires containing the participants' opinions and acoustic analysis.	The teachers reported less fatigue when using the amplifier. The recorded voices, during the use of the amplifier, were considered less tense. In the acoustic analysis, a decrease in spectral tilt was found in the voices that used the amplifier.
6. McCormick et al., USA, 2002.	To examine the effectiveness of the ChatterVox Portable Voice Amplification System (Siemens Hearing Instruments) in reducing the sound pressure level (SPL) of speakers' voices during a simulated lecture in the classroom.	10 speakers.	Read phonetically balanced text using a voice amplifier coupled to sound return boxes. The participant had the auditory feedback amplified for 2 minutes of reading and in the middle of the task the amplification was turned off and the participant read for another 2 minutes. The level of voice adaptation was measured close to the mouth and at the back of the room.	Voice intensity measurements.	There was a mean decrease in vocal intensity at the level of the mouth of 6.03 dB SPL and a mean increase of 2.55 dB SPL at the back of the room. The ChatterVox amplification device reduces the vocal intensity level at the microphone.
7. Jónsdottir et al., Finland, 2002.	To investigate changes in speech during a teacher's working day under normal conditions and when using a voice amplification device.	3 female teachers and 2 male teachers	Teaching during a day of intense work under normal conditions and amplified by a microphone and speaker system calibrated for a maximum signal of 80dB. The recordings were made in the first and last class of the teacher's shift, lasting 40 minutes each.	F0, sound pressure level and questionnaires containing the participants' opinion.	An increase in F0 and sound pressure level was found during the experiment, but the change was greater in F0 when amplification was used. All teachers reported less vocal fatigue when using amplification. The results support the suggestion that an increase in F0 and sound pressure level are not just a sign of vocal fatigue, but may even reflect an adequate adaptation to vocal demand.
8. Jónsdóttir, Iceland, 2002.	Verify whether the use of sound amplification in the classroom has beneficial effects on teachers' vocal production and resistance Determine the negative effects of amplification on the speaker and listener.	33 teachers and 791 students.	Teachers taught with and without voice amplification for one week in each condition. The amplification system was through lapel microphones and the reception through external speakers.	Students' perception and teachers' self-perception.	97% of teachers reported easier voice production, 82% improved vocal endurance. 84% of students found it easier to listen and 63% of students found concentration improved when amplification was used. The negative points reported by teachers and students were technical problems with the devices.
9. Laukkanen et al., Finland, 2002.	Investigate the effects of HearFones through self-perception, auditory and acoustic perceptive analysis on the received intensity, voice quality in singing, vocalization and reading tasks.	Test 1: 2F and 2M. Test 2: 9F, 4 M. Test 3: 6 speech therapists.	Tests: 1 - Text reading sample with and without HearFones. 2 – Singing sample with and without headphones. 3 – Emission of the vowel /pa/, text reading and singing, with and without HearPhones.	Acoustic analysis, electroglottography, auditory perceptive analysis, self-perception of quality and vocal comfort.	HearFones seems to improve voice harmonics, decrease vocal intensity. Participants perceived their voices as “less strained” and “better in control”. Electroglottography indicated better glottic closure and/or decreased activity of the thyroarytenoid muscle while using the device.
10. Roy et al., USA, 2002.	Compare the effects of guidance on vocal hygiene versus voice amplification in teachers with voice problems.	44 teachers with vocal complaints, divided into 3 groups: control group, vocal hygiene group and portable sound amplifier group (Chatter-Vox).	Participants in each group were instructed to use vocal hygiene strategies, amplifier use or no intervention according to their allocation in the group for six weeks.	Self-perception of voice handicap and severity of the voice problem and perception of the strategy used, acoustic and auditory perceptive analysis.	There were no differences between the sound amplification and vocal hygiene groups. The amplification group reported greater clarity and greater ease of voice production with greater adherence to the proposed strategy. The findings support the clinical utility of sound amplification as an alternative for the rehabilitation of vocal problems in teachers.
11. Nsdottir et al., Finland, 2000.	Test whether sound amplification reduces the vocal production load.	5 women.	Reading text of 133 words under normal circumstances, hearing your own amplified voice, through headphones and with auditory feedback dampened by foam earplugs inserted in the external auditory canal.	Acoustic modifications of F0 and sound pressure level.	The F0, sound pressure level and the first formant decreased during amplified and damped feedback. The results suggest that both amplification and damping of auditory feedback can reduce vocal load during phonation.
12.Chang-Yit et al., USA, 1975.	Evaluate the effect and stability of own voice amplification under different amplification conditions over time.	Experiment 1 = 9 college students; experiment 2 = 6 university students.	Experiment 1: spontaneous speech for 12 min while the voice was amplified by 20dB; experiment 2: spontaneous speech for 6 min while the voice was amplified by 20dB or 10dB and speech for 6 minutes while the voice was amplified and 80 dB noise was added. Experiment 2 was repeated for 5 days.	Sound pressure level modifications in voice modification.	The compensatory adjustment in voice in experiment 1 was the reduction from 7 dB to 20 dB of amplification. In experiment 2 the effect was the same in all repetitions of the experiment. The continuous presentation of noise does not desensitize the subject to the effect of inserting noise. The amplification effect is a component of speech regulation.
**Sidetone and Lombard Effects**					
**Author, country, year**	**Objectives**	**Sample**	**Task**	**Variables**	**Main findings and conclusion**
1. Bottalico et al., USA, 2016.	Evaluate the effects on pitch inaccuracy between the reference notes and the note sung under the conditions: 1) level of external feedback, (2) tempo (slow or fast), (3) articulation (legato or staccato), (4) tessitura (low, medium, or high) and (5) semi-phrase direction (ascending or descending).	20 subjects, both sexes, divided into professional and semi-professional singers.	Singing repetitions of arpeggios at different tempos and articulations under the conditions of unaltered feedback, feedback augmented by reflective panels, and feedback diminished by earplugs.	Tuning accuracy.	The inaccuracy was greater when the tempo was faster and the articulation was staccato in semi-professional singers. However, professional singers were more accurate in the diminished feedback condition than in the other external feedback conditions. With increasing training, the inaccuracy of the singer's pitch decreases.
2. Bottalico et al., USA, 2015.	To analyze the Lombard effect, the relationship between sound pressure level and auditory feedback, the relationship between voice quality and external auditory feedback, level of accompaniment, voice register and the singer's gender in professional and non-professional singers.	10 amateur singers and 10 professional singers of both sexes.	Singing excerpts of the same song under the following conditions: unaltered auditory feedback, amplification and reduction of auditory feedback while using a musical accompaniment at three levels (70, 80 and 90 dBA) inserted through headphones.	F0, voice quality.	The Lombard effect was strongest for amateurs, higher levels of external auditory feedback were associated with a reduction in sound pressure level, and this effect was strongest in amateur singers. Better voice quality was detected in the presence of higher levels of external auditory feedback.
3. Bottalico et al., USA, 2015.	Evaluate the effects of voice style (soft, normal and loud), background noise level and external auditory feedback on vocal effort and self-reported vocal comfort, control and vocal fatigue.	20 subjects with no complaints.	Reading a text in a soft, normal and loud way, lasting between 1 and 2 minutes, in a semi-reverberant room with and without panels that increase auditory feedback, and in noise conditions of 40 dB and 61 dB.	Self-perception of fatigue, comfort and vocal control.	Participants increased their level of fatigue in the presence of noise and when instructed to speak in a loud style. They lessened fatigue when feedback was increased and when speaking in a smooth style. In self-perception, there was a preference for the normal style without noise.
4. Siegel and Pick, USA,1995.	Check changes in sound pressure level when there is an increase or decrease in auditory feedback, in the presence or absence of noise under normal or instructed conditions to compensate for changes.	20 individuals of both sexes.	Speak spontaneously while amplifying or reducing the auditory feedback of your voice by 20 dB. First instruction: carry out the necessary compensations for the different types of manipulation. In the second instruction: do not change the sound pressure level in view of the modifications.	Changes in sound pressure level.	The reduction or amplification effect was greater when subjects were instructed to compensate for changes in volume changes. The presence of noise increased the subjects' compensation responses. The presence of noise increases response to auditory feedback manipulations.

Four articles (corresponding to 6.66% of those found) studied the effects of manipulating the intensity, including the Lombard and sidetone effects in the same research; they were cross-sectional experimental studies. The studies used different tasks to verify the effects of amplifying the voice. The populations comprised singers (n = 2) and individuals without vocal complaints (n = 2) of both sexes (n = 4). They used reading (n = 1), speech (n = 1), and singing samples (n = 2). The intensity was manipulated with different resources, including electronic amplifiers, acoustic reflection boards, and feedback systems with earphones and acoustic amplifiers. The variables used in the studies included acoustic analysis and self-perceived comfort. The authors found improved voice quality and F0 control in auditory feedback amplification and increased effort in the presence of noise.

Moreover, 25 studies (41.67% of those found) investigated auditory feedback frequency manipulation (pitch-shift); all of them are cross-sectional experimental studies. In general, they aimed to observe the reflex of pitch shifts and its applications in the study samples. Most samples included both sexes (n = 14), having people without vocal complaints (n = 24) and non-occupational voice users (n = 19). The most used tasks were the emission of sustained vowels (n = 18) and singing (n = 3). F0 variation was the variable most studied (n = 19) to assess the effect, though some studies used other variables to understand the reflex, such as the magnitude and direction of the reflex response, electroencephalography, electroglottography, laryngeal imaging, response time, and cepstral measures. All experiments verified reflex responses to pitch shifts in auditory feedback, although each study had different characteristics (Chart 2).

**Chart 2:** Studies on manipulations of auditory feedback intensity (Pitch-shift-reflex) according to author, publication year, study country, objective, method, and conclusions

**Author, country, year**	**Objectives**	**Sample**	**Task**	**Variables**	**Main findings and conclusion**
1. Larson et al., USA, 2021.	Examine whether the internal voice reference is fixed or variable by comparing f0 with responses to changes in auditory feedback under two presentation conditions.	33 participants (26 F and 7 M).	Vocalize the vowel /a/ in the conditions: auditory feedback altered and introduced during phonation; altered auditory feedback, presented before the beginning of vocalization and removed during vocalization. The modifications were 0.25, 1 and 2 semitones.	Time, magnitude and direction of stimulus response.	There were no differences in response latency or magnitude between time conditions, indicating that for a sustained vowel vocalization task, the internal referent is not fixed.
2. Alem et al., Canada, 2021.	Testing the hypothesis: the duration of adaptive responses to changes in auditory feedback and the adaptation to the time spent on each emitted frequency depends on the task performed, be it singing, reading or vocalizing.	30 participants (16 F and 14 M).	Sing “Happy Birthday”, read a paragraph from Harry Potter and vocalize /a/, /e/, /o/ with and without a 1 semitone shift in auditory feedback.	Tuning Analysis and F0 Modifications.	The adaptive motor commands used by individuals with normal hearing are malleable through changes in feedback, perhaps more so when reading aloud than when singing or vocalizing. But these effects are revealed through subtle changes in voice pitch variations.
3. Kothare et al., USA, 2020.	To test how much the adaptation response opposes the auditory feedback change and how much it varies depending on the direction of the feedback change applied to the vowel formants.	18 participants (10 F and 8 M).	Speak preselected words while the frequency of the first and second formants (F1 and F2) are changed up to 50 Hz up and down.	Acoustic measurements.	Adaptation takes place depending on the direction of displacement applied in the space of the vowel formant, regardless of the magnitude of the displacement.
4. Alexandra Schenck et al., USA, 2020.	Evaluate the relationship between auditory feedback control and voice quality measured by smoothed cepstral peak prominence (CPPS), reflected in the voice signal harmonics.	25 healthy adults.	Produce sustained vowels while the auditory feedback is modified in intensity (0, 3 or 6 dB) and frequency (0, 50 or 1 semitones).	Smoothed cepstral peak prominence (CPPS).	The increase and decrease in intensity caused a relative increase in CPPS, indicating an improvement in voice harmonics, even in cases where vocal intensity was reduced. Results indicate that there is a voice quality control mechanism that increases the harmony of the voice signal to improve audibility in the presence of unpredictable variability in loudness.
5. Behroozmand et al., Germany, 2020.	To investigate how high definition transcranial direct current stimulation of the left ventral motor cortex modulates the neural mechanisms of sensorimotor integration during voice motor control.	30 participants (20 F and 10 M).	Vocalize sustained vowel /a/ with 1 semitone variations in up and down auditory feedback.	High definition transcranial continuous current stimulation of the left ventral motor cortex at two currents (1 mA or 2 mA).	There is no differential modulation effect of 1 mA versus 2 mA. Neurostimulation of the left ventral motor cortex modulates the sensorimotor mechanisms controlling the underlying voice motor.
6. Hilger et al., USA, 2019.	To investigate how the direction and timing of a disturbance in the auditory feedback of pitch of voice during sentence production modulate the magnitude and latency of the pitch change reflex.	32 participants (21 F and 11 M).	Produce three sets of phrases while applying pitch perturbations (2 semitones higher or lower) to the auditory feedback through headphones on the first, second, or third word of each phrase.	Voice acoustics and f0 variations.	The pitch change reflex was greater after disturbances in the first word of the sentence. End-of-sentence word production was acoustically improved after disturbances at the beginning of the sentence, but even more so after disturbances of the first word of the sentence. Participants can integrate feedback-based error-correcting commands by revising acoustically-related early intonation target motor plans into phrasal production.
7. Ziethe et al., Germany, 2018.	To analyze the functionality of phonation and speech control mechanisms between patients with muscle tension dysphonia (TMD) and normal individuals.	61 healthy individuals and 22 with TMD.	Sustained phonation /a/ and speech with auditory feedback altered by 7 semitones down or up, input through headphones.	Electroencephalography, electroglottography, acoustic voice signal and video signal.	There were changes in both groups between the “no pitch” and “pitch” condition of the two conditions in relation to vocal fold dynamics and voice quality. TMD patients showed more vibratory irregularities during feedback modification than controls. TMD patients seem to have a disturbed interaction between auditory and kinesthetic aspects.
8. Alsius et al., USA, 2017.	Test whether speech target modifications have an impact on the fine-tuning of vocal motor commands.	64 women.	Producing the word “head” while the received auditory feedback was altered by systematically changing the first formants of the vowel /e/ (up to 200 Hz) in real time through headphones, while inserting linguistic and non-linguistic prompts for correction.	Fundamental frequency modifications.	Linguistic commands induced greater corrective behavior for acoustic disturbances than non-linguistic commands. The automatic correction of vocal adaptations is influenced by flexible and context-dependent mechanisms.
9. Arbeiter et al., Germany, 2017.	To investigate changes in auditory feedback mechanisms and voice quality during phonation in response to a spontaneous pitch change in auditory feedback.	28 participants.	Phonation of the vowel /a/ listening to the auditory feedback altered in 7 semitones received by headphones.	Electroencephalography (EEG), acoustic voice signal, electroglottography (EGG), and high-speed videoendoscopy (HSV).	The tone change reflex was successfully detected in all variables used. A significant increase in disturbance measures and an increase in acoustic parameter values ​​during pitch change were observed, mainly for the audio signal. The auditory feedback mechanism seems to control not only the pitch of the voice, but also the quality of the voice.
10. Petermann et al., Germany, 2016.	Allow detection of Pitch Change Reflex providing detailed analysis of kinesthetic feedback in future work.	5 participants (2 M and 3 F).	Producing the vowel /a/ and the syllables /mama/ while the auditory feedback of the voice was changed by 7 semitones down or up inserted into headphones.	Voice acoustic analysis, electroencephalography, electroglottography and laryngeal images.	Pitch change reflex was found in physiological latency intervals for EEG, EGG and signals in voice acoustics. It was also successfully verified in the laryngeal dynamics data, obtained by laryngeal images, which showed similar sensitivity as EGG and voice signals.
11. Behroozmand et al., USA, 2015.	To investigate the motor control mechanisms of pitch of voice, examining the spectro-temporal dynamics of EEG signals in non-musicians (NM), musicians with relative pitch (RP) and musicians with absolute pitch (AP) during pitch change.	34 subjects (11 non-musicians, 12 musicians with relative pitch, 11 absolute pitch).	Maintain vowel vocalizations /a/ while receiving pitch shift stimuli of 1 semitone down and up in their auditory feedback inserted by headphones.	Spectro-temporal dynamics of EEG signals.	Delta activation was significantly stronger in NM. Evoked theta is a neurophysiological marker of enhanced pitch processing in musicians and reflects mechanisms by which humans incorporate auditory feedback to control the pitch of their voice. Delta activation reflects adaptive neural processes by which vocal production errors are monitored and used to update the state of sensorimotor networks for driving subsequent vocal behaviors.
12. Patel et al., USA, 2015.	Investigate possible automatic mechanisms that may be involved in voice frequency control at register limits.	9 singers (6 F and 3 M).	Singing notes at the ends of registers while the pitch of the voice's auditory feedback is unexpectedly changed to the adjacent register or within the modal register. Changes were entered by headphones.	Changes of F0 and electroencephalography.	Singers adapt to the sudden shift to the basal register by activating neural mechanisms that can lessen the magnitude of a change in voice quality.
13. Flagmeier et al., USA, 2014.	Use the structural equation model and functional neuroimaging data to examine neural properties of a voice with and without modified auditory feedback.	10 subjects (4 M and 6 F).	Vocalize vowel /a/ for 5 seconds interspersed with rests, while the auditory feedback, inserted through headphones, is altered by 1 semitone downwards or upwards during phonation.	Structural equation model and functional neuroimaging data.	The presence of a pitch change, which was processed as a vocalization error, was recorded as altered connections between the right and left superior temporal gyrus, the latter playing a role It plays an important role in detecting and correcting errors. The results suggest that the right hemisphere is fundamental for pitch modulation.
14. Korzyukov et al., USA, 2012.	Understand sensorimotor integration during vocalization, speech and its complex components.	10 participants (8 F and 2 M).	Vocalize the vowel /a/ in the usual tone while the auditory feedback was altered by 1 or 4 semitones up and down. 100 samples were collected with and without feedback changes.	F0 changes, electroencephalography, dynamic causal modeling and event-related potentials.	The results suggest that both the intrinsic superior temporal gyrus and left-to-right connections are important in identifying voice changes and sensorimotor integration. Own voice modifications and non-own voice modifications are processed differently in the right and left hemispheres.
15. Behroozmand et al., USA, 2012.	Analyze vocal responses to pitch disturbances in voice feedback in which correction attempts are classified according to response direction and averaged into groups of ascending or descending responses.	15 participants (10 F and 5 M).	Vocalization of the vowel /a/ in his usual speaking tone while the feedback frequency was changed by 1, 2 or 5 semitones up and down in a total of 25 vocalizations.	Variations of F0.	The predictability of stimulus direction and magnitude can modulate vocal responses to feedback tone disturbances.
16. Behroozmand et al., USA, 2011.	To investigate the neural mechanisms of voice tone control for different stimulus modifications in auditory feedback.	12 participants (6 M and 6 F).	Sustained vocalizations of the vowel /a/ while auditory feedback was modified by 2 semitones upwards. The types of feedback were: 1. Your own voice; 2. A pure sinusoidal tone at the fundamental frequency of your own voice; 3. tones with F0 and its first harmonic frequency; 4. F0 with its first and second harmonic frequencies; 5. F0 with its first, second and third harmonics. Changes entered through headphones.	Acoustic changes, electroencephalogram data.	During active vocal production, pitch change reflex amplitudes were greatest in response to pitch changes in the natural voice, moderately large for complex non-vocal stimuli, and smallest for pure tones. During passive listening, neural responses were equally large for pitch changes in voice and non-vocal communication with complex stimuli, but even greater than for pure tones.
17. Liu et al., China, 2011.	To investigate age-related changes in auditory voice feedback control during sustained vocalization. Understand how F0 vocal responses vary throughout adulthood and at what age people will produce vocal responses that differ from those produced by young adults.	60 individuals of both sexes divided into 5 age groups.	Sustaining vowel /u/ while pitching auditory feedback is changed by 6.50 or 61 semitones up and down inserted into headphones.	F0 changes and latencies in pitch changes.	Response magnitudes increased with increasing age until maximum values ​​were reached for adults aged 51-60 years and then decreased for adults aged 61-75 years. Adults aged 51 to 60 years were also more sensitive to the direction and magnitude of pitch feedback perturbations compared to younger adults. The pitch shift reflex changes throughout adulthood.
18. Larson et al., USA, 2008.	Test the hypothesis that the elimination of kinesthesia would be associated with a greater response to an external auditory disturbance.	19 individuals (9 F and 10 M).	Vocalize the vowels /u/ and /i/ with auditory feedback modification by 5 and 1 semitones up and down in conditions with and without vocal fold anesthesia.	Fundamental frequency modifications.	Vocal fold anesthesia increases the response to an externally imposed auditory disturbance. There are differences for these sensory channels: auditory feedback can be used for full F0 control while kinesthesia is used when auditory feedback is not available.
19. Jones et al., Canada, 2008.	Examine the differences of modifications between F0 feedback and the vocal production system in singers and non-singers.	40 participants (20 singers and 20 non-singers).	Singing the syllable /ta/ while auditory feedback was shifted down 1 semitone.	Average values ​​of F0.	Singers rely more on internal models than non-singers to regulate vocal productions. Non-singers need more real-time auditory cues.
20. Sivasankar et al., USA, 2005.	Examine whether a subject's F0 responded not only to perturbations in the pitch of voice feedback, but also to changes with other presented pitch stimuli congruent with the voice feedback.	19 healthy women.	Vocalizing the vowel /u/ in a steady habitual tone while feedback was modified 1 semitone higher or lower through headphones under the conditions: 1. Return of own voice. 2. Pure Tone Return. 3. Return of own voice and pure tone.	Changes from F0.	Subjects responded to F0 changes rather than pure auditory feedback tones. The audio-vocal system is sensitive to changes in the pitch of a variety of sounds, which may represent a flexible system capable of adapting to changes in the subject's voice. This system can reduce the influence of other sounds when you have feedback from your own voice.
21. Leydon et al., USA, 2003.	Demonstrate that properties of the auditory system support vibrato by initiating pitch-changing reflex responses as subjects produce a continuous tone.	6 participants (5 F and 1 M).	Sustaining the /E/ vowel steadily while the auditory feedback is modulated 0.25 semitones up and down.	Changes and oscillations of f0 and frequency transfer functions.	Transfer functions revealed peak gains at 4 to 7 Hz in all subjects, with an average peak gain at 5 Hz. These gains occurred in frequency in regions where voice output and auditory feedback signals were in phase. A control circuit in the auditory system can sustain vocal vibrato and tremor-like oscillations in the voice
22. Burnett et al., USA, 2002.	Examine whether the initial component of the pitch-changing response is unique to constant-pitch vocalizations, or whether it is a mechanism that could help control pitch-stable and dynamic vocalizations.	30 professional singers.	Sustained vocalizations of the vowel /a/ and glissando while the pitch of the auditory feedback was shifted up by 1 semitone and inserted with headphones.	Fundamental frequency modifications.	Pitch-shifting responses occurred during glissando vocalizations. These responses have higher latency and lower magnitude than the responses during stable note phonation. This response serves to automatically bring the phonation tone according to an intended target, whether this target is constant or not.
23. Liu et al., China 2002.	To investigate the effect of stimulus timing on vocal responses to pitch-changing feedback on different intonation patterns during Mandarin speech production.	10 participants.	Speaking a sentence during the conditions: 1. fundamental frequency (f0) of the final word increased (question intonation); 2. fundamental frequency (f0) of the final word slightly decreased (statement intonation) or change of 1 semitone in the feedback presented at three different moments (160, 240 or 340 ms) after the beginning of the vocalization.	Modifications of f0 and response latency.	Response magnitudes were reduced for the 340 ms condition compared to 160 or 240 ms for intonations. A planned change in F0 can cause a modulation in the reflexive response. There is a critical time period during which response mechanisms are most sensitive to the planning process.
24. Jones et al., Canada, 2000.	Present data that address the role played by acoustic feedback in voice F0 control.	18 men.	Produce vowel /a/ on feedback with F0 normal, F0 shifted up and F0 shifted down by 1 semitone	Modifications in F0.	Subjects compensated for the change in F0. When the F0 feedback returned to normal, the subjects modified their F0 producing the opposite direction of change. Results suggest that F0 is controlled through auditory feedback and with reference to an internal pitch model.
25. Burnett et al., USA, 1997.	To analyze changes in F0 during a pitch change in auditory feedback in a group of normal subjects and a small number of trained singers.	67 participants of both sexes (15 singers and 52 non-singers).	Emitting the vowel /a/ and singing scales ignoring the different frequency modulations up and down (0.25; 0.5; 1; 1.5; and 2 semitones) that were presented in the auditory feedback.	Modifications in f0.	96% of subjects increased their F0 when pitch feedback was decreased, and 78% decreased their F0 when pitch feedback was increased. Results indicate that people rely on auditory feedback to control F0 voice.

## DISCUSSION

This scoping review mapped the literature available on auditory feedback manipulations in adults’ voice motor control. Many articles have addressed the different manipulations. However, most authors focused on studying auditory feedback suppression (Lombard effect)^(23-37)^ and pitch manipulation with the pitch-shift reflex effect^(5,38-60)^.

There is a need for more in-depth studies on the multidimensional effects of auditory feedback amplification on the voice. Each study analyzes a type of outcome, but there are few elements to understand the proportions between auditory feedback amplification and decreased voice intensity.

The studies that researched the Lombard effect had different samples, including adult singers, teachers/professors, healthy individuals, reporters, dysphonic individuals, and musicians. Regardless of the population, most results involved increased voice production intensity in the various tasks^(23-25,27,31,33,34,61,62)^. Few studies observed feedback suppression in dysphonic subjects. Studies have shown that this population is seemingly more sensitive to the effects of auditory feedback suppression and find it more difficult to return to the habitual speech intensity when the noise is removed^(25,31,34,63)^.

Concerning outcome variables, most studies on the Lombard effect addressed the changes in voice intensity and frequency^(23,24,26-28,31-34,36,37,63,64)^. Some of them also included vocal dose^(27,31)^, auditory-perceptual evaluation^(29)^, laryngeal resistance^(30)^, aerodynamic measures^(30,65)^, laryngeal assessments, vowel duration, formant means^(10,24,65)^, magnetic resonance, and electroencephalography^(25,28)^.

There is no consensus on the intensity of the masking noise to induce the Lombard effect. Some researchers used signals other than noise via earphones to mask the auditory input, such as music and external noise^(26,31,37)^. The studies used different intensities, ranging from 40 to 100 dB; the one most used in the methods within this range was 90 dB^(29,33,63)^. Some studies did not establish a fixed intensity; rather, they were only based on each participant’s threshold^(23,32)^. This shows the need for researching which minimum intensity triggers the Lombard effect and what are the differences between populations, as the literature reports a proportion observed between the noise level and the voice amplification level^(18)^.

Concerning the main findings of the Lombard effect, most studies state that decreasing one’s own voice auditory feedback leads them to unconsciously increase their vice intensity^(10,23-30,34,35,63,65)^, corroborating the literature on the effect^(11,12,18)^. In general, the authors found increased vocal doses, increased voice intensity, imprecise pitch correspondence, and greater susceptivity to the effect in strained voices^(25-27,30,31,36,63,65)^.

Furthermore, the studies observed that the level of vocal change depends on each person^(29,31)^, which indicates a difficulty in generalizing it and finding a proportion between noise intensity and voice production intensity. Another interesting finding is that individuals can control the changes caused by auditory feedback suppression, based on simple orders or visual feedback^(37,63)^.

Another conclusion is that the Lombard effect in beginner singers diminishes tuning precision in complex tasks. Hence, it is inferred that these singers need their hearing to correspond to pitches indicated in complex tasks, whereas more advanced singers do not depend so much on auditory feedback to master their tuning, as previously demonstrated in the literature^(25,27)^ – reporters likewise^(31,36,62)^.

It is hypothesized that training kinesthetic skills helps control voice intensity with less influence from auditory feedback. On the other hand, studies on dysphonic patients have shown that they tend to respond in greater magnitude to the effect^(24,30)^.

The researchers of studies on pitch-shift effect feedback included variables such as electroencephalography, spectro-temporal dynamics, and functional neuroimaging^(41,45-49,51)^, which verify cortical activities, and associate them between and within hemispheres related to reflex pitch shifts to understand in-depth the response to changes in frequency in auditory feedback^(41)^. Some studies analyzed neuroimaging and electroencephalography^(49)^ and described that the right hemisphere plays an essential role in pitch modulation.

The articles describe changes when the feedback goes 0.25 to 7 semitones up or down. However, the minimum manipulation level to obtain a reflex response has not yet been defined^(17,49-60)^. All experiments used hardware and software that change frequencies and earphones to present the modified signal to participants. Many selected articles on the topic used 1-semitone changes in their experiments^(7,17,40,41,47,49,50,53-55,58,59)^.

The tasks varied considerably, including different vowels sustained in constant pitches, corroborating the literature that described the pitch-shift reflex in this task^(10,15-17)^. Some experiments used singing tasks^(38,48,54,59)^, or the emission of syllables^(46,54)^, words, and sentences^(38,42,44,58)^ as the frequency is displaced in auditory feedback. The sustained vowels used are not standardized; the most recurrent ones are /a/ and /u/^(17,38,41,43,45-47,49-56)^, though some articles used other ones^(38,45,51,54,56)^. The studies have found a correction reflex to the manipulation of the auditory feedback frequency – i.e., if the feedback goes up, participants tend to correct it by decreasing the production frequency, and vice-versa, as described in other articles^(15-17,24)^.

The literature available describes different results between vocally healthy and dysphonic populations – the latter seems to have a greater reflex response to pitch changes in auditory feedback^(42)^. However, no quantitative or proportional pattern has been found yet regarding such changes triggered by manipulation among dysphonic and non-dysphonic individuals. Studies have concordantly observed that singers rely more on their internal tuning model than non-singers. The longer the training in singing tasks, the more signers tend to rely on their internal tuning model, rather than on the auditory feedback. The manipulations did not have the same magnitude in trained and untrained singers^(47,48,54,57,59)^. These data point to the hypothesis that trained individuals have well-established internal models, little influenced by external changes and updates, in contrast with individuals without training or with vocal changes.

The sidetone effect was tested with spontaneous speech and reading tasks in different study populations, with greater emphasis on teachers/professors^(66-71)^, as they are constantly subject to the Lombard effect with great vocal demand^(64,72,73)^. Voice intensity was the most used outcome, which was expected to reduce with amplification^(61,63,69,72,74)^. Other variables included the subjects’ self-perception and the auditory-perceptual evaluation of voice^(60,68-71,74)^.

The studies do not focus on a specific amplification system. They used portable amplifiers^(67,68,71,73)^, loudspeakers^(63,69,70,75)^, and feedback earphones^(60,74,76)^. The findings show that all experiments described a decrease in vocal loading and effort when the own voices were amplified, demonstrated in the sound pressure level and self-perceived effort^(60,61,63,66-71,74,75)^. All studies are conclusive about the response of decreasing voice production intensity, as previously described in the literature^(60,61,63,66-71,74,75)^. Some of them also describe changes in the voice frequency, agreeing that the voice is better controlled when it is amplified^(71,74,77)^.

The studies also report positive voice production results, with less self-perceived phonatory effort^(68,71,74)^. However, each experiment used a different method, and even though auditory feedback voice amplification is described as a therapeutic resource that optimizes speech therapy, no research was found addressing its effects along with voice therapy.

Equipment used also varied, and there are no comparisons of the effectiveness obtained with the different forms of using the same type of manipulation. Likewise, few studies observed feedback changes and manipulations in dysphonic individuals. Hence, effectiveness studies are needed to verify the results of using amplification in vocal rehabilitation^(66,67,71)^. These gaps in the literature are fields of research that still need scientific exploration.

The studies in this scoping review show that the levels of amplification are not standardized and that there is no conclusive value of the decrease in voice production intensity in response to specific values of auditory feedback amplification. Few studies explore intensity proportions or levels in their experiments^(61,70)^. This gap makes it difficult to establish a contrary relationship or correlation of the proportion found in the Lombard effect^(18)^.

On the other hand, experiments that associated the effects of auditory feedback suppression and amplification also had different objectives, such as assessing task precision in singers and verifying comfort levels and sound pressure levels in normal individuals^(61,77-79)^. This review did not find standardized levels of amplification or noise insertion, as studies used different experiment methodologies and tasks (singing and text reading tasks).

This scoping review explored experiments and condensed research results with auditory feedback manipulation for adults’ speech-motor control. However, the methodological diversity between experiments, sometimes with scarce information, unstandardized speech tasks, different outcome variables, and small samples may have limited the results. Nevertheless, this review pointed out gaps in current knowledge, encouraging further research on the topic, and, therefore, helping increase the knowledge of voice training or therapy.

## CONCLUSION

Mapping the current literature on experiments with auditory feedback included in this scoping review shows that different methods are used to amplify, suppress, and manipulate auditory feedback frequency. Results in general are similar regarding the reflex response in voice motor control, observing each experiment’s specificities. However, the relationship between the magnitude of manipulation and the responses still needs to be better understood. The main findings in research on auditory feedback for voice motor control demonstrate that, in auditory feedback suppression, individuals tend to increase their voice intensity. In auditory feedback amplification, they decrease voice intensity and have greater control over F0. In frequency manipulations, they tend to correct the manipulation. The few studies that conducted experiments in dysphonic individuals showed that they behave differently from non-dysphonic subjects.
