# Supplementary material for: Modifications of auditory feedback and its effects on the voice of adult subjects: a scoping review
Source: Codas. 2023 Dec 22;36(1):e20220202. doi: 10.1590/2317-1782/20232022202en (PMC10750862; doi:10.1590/2317-1782/20232022202en)
Supplement: Translation

# Translation: Modificações do feedback auditivo e seus efeitos sobre a voz de indivíduos adultos: uma revisão de escopo

**DOI:** 10.1590/2317-1782/20232022202pt

**Authors:** Moisés do Carmo Alves, Patrícia Cotta Mancini, Leticia Caldas Teixeira

**Affiliations:** 1 Programa de Pós-graduação em Ciências Fonoaudiológicas, Departamento de Fonoaudiologia, Faculdade de Medicina, Universidade Federal de Minas Gerais – UFMG - Belo Horizonte (MG), Brasil.

**Keywords:** Feedback Auditivo, Percepção Auditiva, Retroalimentação, Controle Motor da Voz, Indivíduos Adultos, Estudo de Revisão

## Abstract

**Introdução:**

A percepção auditiva da voz e sua produção envolvem o feedback auditivo, as pistas cinestésicas e o sistema de feedforward, os quais produzem efeitos distintos para a voz. Os efeitos Lombard, Sidetone e o Pitch-Shift-Reflex são os mais estudados. O mapeamento de experimentos científicos sobre as modificações do feedback auditivo para o controle motor da voz possibilita examinar a literatura existente sobre o fenômeno e pode contribuir para o treinamento ou terapias da voz.

**Objetivo:**

Mapear os experimentos e resultados das pesquisas com manipulação do feedback auditivo para o controle motor da voz de indivíduos adultos.

**Método:**

Revisão de escopo seguindo o Checklist Preferred Reporting Items for Systematic reviews and Meta-Analyses extension (PRISMA-ScR) para responder à pergunta: “Quais os métodos de investigação e principais achados das pesquisas sobre a manipulação do feedback auditivo no automonitoramento da voz de indivíduos adultos?”. O protocolo de busca foi baseado na estratégia mnemônica População, Conceito e Contexto (PCC). A população são os indivíduos adultos; o conceito é a manipulação do feedback auditivo e o contexto é o controle motor da voz. Os artigos foram pesquisados nas bases de dados: BVS/ Biblioteca Virtual em Saúde, MEDLINE/Medical Literature Analysis and Retrieval Sistem on-line, COCHRANE, CINAHL/Cumulative Index to Nursing and Allied Health Literature, SCOPUS e WEB OF SCIENCE.

**Resultados:**

Foram encontrados 60 artigos, sendo 19 da temática do Efeito Lombard, 25 do efeito Pitch-shift-reflex, 12 do efeito Sidetone e quatro sobre o efeito Sidetone/Lombard. Os estudos são concordantes que a inserção de um ruído que mascara o feedback auditivo provoca um aumento na intensidade de fala do indivíduo e que a amplificação do feedback auditivo promove a redução do nível de pressão sonora na produção da voz. Observa-se uma resposta reflexa à mudança de tom no feedback auditivo, porém, com características individuais em cada estudo.

**Conclusão:**

O material e método dos experimentos são distintos, não há padronizações nas tarefas, as amostras são variadas, muitas vezes reduzidas. A diversidade metodológica dificulta a generalização dos resultados. Os principais achados das pesquisas a respeito o feedback auditivo sobre o controle motor da voz confirmam que, na supressão do feedback auditivo, o indivíduo tende a aumentar a intensidade da voz. Na amplificação do feedback auditivo, o indivíduo diminui a intensidade e tem maior controle sobre a frequência fundamental e, nas manipulações da frequência, o indivíduo tende a corrigir a manipulação. Os poucos estudos com sujeitos disfônicos mostram que eles se comportam diferentemente dos não disfônicos.

## INTRODUÇÃO

A audição exerce grande importância na produção e automonitoramento da voz e sua influência sobre a produção vocal tem sido explorada na literatura científica^(1-7)^. A produção da voz e seu monitoramento envolvem três mecanismos: o *feedback* auditivo, as pistas cinestésicas ou *feedback* somatossensorial e o sistema de *feedforward* ^(7)^.

O *feedback* auditivo é a percepção auditiva da própria voz em tempo real que permite monitorar a intensidade, a frequência e qualidade da própria voz^(5,8-10)^. O *feedback* somatossensorial se relaciona à percepção das adaptações e aos ajustes motores das estruturas envolvidas no processo da fonação^(11)^.

Os feedback*s* auditivos e somatossensoriais contribuem para produzir referências internas para o planejamento motor da fala, e realizar atualizações desses ajustes para o sistema *Feedforward* ^(9)^. Esse sistema é descrito, teoricamente, como um sistema cortical, localizado no hemisfério esquerdo do cérebro, responsável pelo mapeamento dos movimentos articulatórios dos lábios, mandíbula, língua e laringe, e que armazena esses ajustes motores de fala à partir de referências motoras, somatossensoriais e auditivas^(8)^. O sistema Feedforward usa dessas referências internas, adquiridas previamente, para controlar a voz^(7)^.

Diferentes tipos de manipulação no *feedback* auditivo produzirão efeitos distintos para a voz do indivíduo, sendo o efeito Lombard, efeito *Sidetone* ^(12-14)^ ou de amplificação, e o efeito *Pitch-Shift* os mais estudados^(15-18)^.

O efeito Lombard acontece quando há o aumento da intensidade da produção voz, mediante a inserção de um ruído intenso, que mascara o *feedback* auditivo. A ação de aumento da pressão sonora da voz é realizada de forma inconsciente e instantânea e, ao se retirar o ruído, o indivíduo vocalmente saudável tende a retornar ao mesmo nível de intensidade da fala anterior à inserção do ruído^(2,18)^.

O efeito *Sidetone* é a amplificação do retorno auditivo que aumenta a percepção do indivíduo em relação à sua própria voz^(12)^. Em resposta a essa manipulação, o indivíduo vocalmente saudável reduz o nível de pressão sonora que produz sua voz^(12)^. O efeito reduz a pressão sonora na voz de pacientes com disfonias hiperfuncionais e cria ou aumenta a percepção auditiva do sujeito quanto aos parâmetros e alterações de sua voz^(8)^, o que lhe permite monitorar a frequência fundamental da voz, bem como sua qualidade e intensidade^(8,12-14)^.

O efeito *Pitch-Shift* acontece quando se expõe auditivamente o indivíduo que não apresenta alterações vocais a uma mudança de frequência da própria voz. Essa manipulação provoca uma alteração reflexa de correção e sabe-se que a reação mais comum é a correção dessa frequência no sentido oposto da manipulação. Outra possibilidade, menos comum, é a modificação no mesmo sentido que ocorreu a manipulação^(10,15-17)^.

Em consequência das modificações vocais produzidas pelas diferentes manipulações do feedback auditivo, faz-se essencial compreender sua utilização no treinamento e na terapia da voz. Sabe-se pouco a respeito dos experimentos em relação ao tempo de exposição ao feedback, nível de amplificação ou de ruído mascarante e a efetividade dessas manipulações para a voz de indivíduos com e sem queixas vocais, profissionais ou não da voz. Acredita-se que mapear a literatura acerca da questão, por meio de uma revisão de escopo, possibilitará examinar a literatura científica existente sobre o fenômeno e verificar suas lacunas, vislumbrando possibilidades futuras para novos estudos.

Diante do exposto, formulou-se a pergunta: “Quais os métodos de investigação e principais achados das pesquisas sobre a manipulação do *feedback* auditivo no automonitoramento da voz de indivíduos adultos? ”

## MÉTODO

Trata-se de uma revisão de escopo que seguiu o detalhamento proposto pelo *Checklist Preferred Reporting Items for Systematic reviews and Meta-Analyses extension para revisões de escopo (PRISMA-ScR)* contidas no *Joanna Briggs Institute Reviewers’ Manual* ^(19)^. Esse *checklist* contém 22 itens que direcionam a redação do relato para a revisão de escopo. A revisão foi realizada entre novembro de 2021 a novembro de 2022. O protocolo de revisão foi registrado em 29 de novembro de 2021 no Open Science *Framework* (OSF) doi:10.17605/OSF.IO/CYM9N.

Foi utilizada a estrutura metodológica de Arksey e O’Malley^(20)^ com as emendas feitas por Levac et al.^(21)^ e por Peters et al.^(22)^: 1) identificação da questão e objetivo de pesquisa; 2) identificação de estudos relevantes; 3) seleção de estudos; 4) mapeamento dos dados; 5) seleção das evidências; 6) apresentação dos resultados.

A seleção, inclusão e extração de dados foram realizadas por dois pesquisadores, de forma independente, quanto à pertinência ou não da seleção e inclusão no estudo. Estabeleceu-se os critérios de inclusão e exclusão, conforme o checklist PRISMA-ScR para revisões de escopo:

Critérios de inclusão: artigos que apresentam o termo ‘*feedback* auditivo’ no título e no resumo; artigos que contemplam a temática implícita no resumo e que se relacionam com o controle motor da voz em indivíduos adultos, profissionais e não profissionais da voz; em tratamento ou treinamento de voz. Artigos escritos nos idiomas português, inglês ou espanhol, sem limite de ano. Esses critérios foram utilizados com o objetivo de rastrear toda a literatura disponível sobre o tema.

Critérios de exclusão: os artigos que utilizavam o *feedback* auditivo em outros contextos, que não o de amplificação sonora da própria voz (efeito *sidetone*), supressão do *feedback* auditivo (efeito Lombard), manipulação da frequência da voz (efeito *Pitch-Shift)*, os que utilizaram o *feedback* com “sons-alvo externos e os artigos que apresentavam populações com acometimentos neurológicos. Artigos que traziam dúvida em relação ao tema, no título ou no resumo, foram buscados na íntegra. Em caso de divergência, os revisores realizaram análise conjunta para definir por consenso a permanência ou retirada de artigos.

### Estratégia de pesquisa - Questão de pesquisa e critérios de busca

A revisão envolveu a formulação de uma pergunta de pesquisa, baseada na estratégia mnemônica PCC, que corresponde a *Population* (População), *Concept* (Conceito), e *Context* (Contexto)^35,38.^ Nesta pesquisa, população são os indivíduos adultos, o conceito é a manipulação do *feedback* auditivo e o contexto é no controle motor da voz.

Como fontes de evidência, utilizou-se os estudos científicos publicados até novembro de 2022 que consideraram o uso do *feedback* auditivo incluindo todo e qualquer tipo de evidência. Os artigos foram pesquisados nas bases de dados: BVS/ Biblioteca Virtual em Saúde, MEDLINE/*Medical Literature Analysis and Retrieval Sistem on-line*, COCHRANE, CINAHL/*Cumulative Index to Nursing and Allied Health Literature*, SCOPUS e *WEB OF SCIENCE*. Para o conceito de “*feedback* auditivo”, utilizou-se os descritores DeCS/MeSH: Percepção auditiva, *Audio feedback* e como palavras chave, os termos: *feedback* auditivo, *Auditory, External auditory, Internal auditory, Kinesthetic, Portable amplification, sound amplification, Auditory masking, Auditory self-monitoring, Sidetone, Pitch-shift auditory, frequency, Shifted, Lombard Effect*. Para o conceito “controle motor da voz” utilizou-se os descritores: Qualidade de voz, *Voice Training, Voice* e como palavras chave os termos: Controle motor da voz, *Voice Control, Pitch Control, Control of voice intensity.*

A Figura 1 mostra a estratégia PCC de busca que foi elaborada pelo primeiro pesquisador e revisada pelo segundo pesquisador. Após a busca, uma pesquisa manual também foi realizada nas referências entre os artigos encontrados a fim de complementar os dados.

**Figura 1:** Elaboração da estratégia de busca

### Mapeamento dos dados

Após a seleção dos estudos, estes foram exportados para a plataforma *Rayyan* – *Intelligent Systematic Review*. A seleção dos estudos foi feita de forma cega por cada pesquisador. A entrada dos estudos conflitantes foi definida entre os pesquisadores por consenso e, após essa seleção, foi discutido e atualizado o formulário de acordo com os dados que julgaram relevantes. Os estudos foram agrupados pelos tipos de manipulação do *feedback* auditivo observando os delineamentos, populações, as tarefas realizadas nos experimentos e as variáveis dependentes utilizadas para mensurar os resultados. Os artigos replicados foram eliminados e os dados foram extraídos e organizados em planilhas, de acordo com o tipo de *feedback*, contendo as informações dos estudos relevantes para essa revisão: autor, país, ano, objetivos, método, principais resultados e conclusão.

### Seleção das evidências

Após a categorização dos estudos, foi realizada uma sumarização dos resultados encontrados em dois quadros, com o objetivo de facilitar a comparação das informações.

## RESULTADOS

A síntese dos resultados das fases de identificação, seleção, elegibilidade e inclusão estão descritos no organograma representado na Figura 2.

**Figura 2:** Diagrama de fluxo da busca na literatura e inclusão de artigos

Foram encontrados 19 estudos, correspondendo a 31,65%, que utilizaram o efeito Lombard. Todos os estudos eram de delineamento transversal. As amostras eram compostas, em sua maioria, por participantes de ambos os gêneros masculino e feminino (n = 15), não profissionais da voz (n = 16) e sem queixas vocais (n = 15). Apenas um estudo incluiu indivíduos com disfonia e cinco estudos foram realizados com cantores. Entre as tarefas, destaca-se o uso de vogais, seguida de tarefas de canto e de tarefas de leitura, respectivamente. A inserção do ruído mascarante variou de 50 a 105 dB e não há consenso ou padronização da medida de ruído. As variáveis de desfecho mais estudadas foram as medidas acústicas, principalmente de F0. Os resultados concordam que a inserção de um ruído que mascara o *feedback* auditivo provoca um aumento na intensidade de fala do indivíduo. Os artigos que pesquisaram adultos cantores concordam entre si que o *feedback* auditivo, mesmo em proporções diferentes, de acordo com o nível de treino, contribui para a precisão da afinação no canto. Indivíduos com problemas de voz parecem se comportar de forma diferente, mostrando dificuldade de retornar ao ajuste usual, após exposição ao ruído mascarante

Doze artigos, 20% dos encontrados, pesquisaram o efeito *Sidetone* (efeito de amplificação) no feedback auditivo (Quadro 1). Onze estudos são do tipo experimental transversal e um trata-se de ensaio clínico randomizado. A maioria dos estudos teve como foco o estudo dos efeitos da amplificação sobre a voz humana. As amostras eram em sua maioria, formada por participantes de ambos os gêneros, masculino e feminino (n = 6) profissionais da voz (n = 7), sendo a maioria deles professores (n = 6). Havia três estudos com sujeitos disfônicos e um com e sem queixas vocais. As tarefas mais utilizadas foram a de fala espontânea (n = 8) seguido de leitura de texto (n = 3). Apenas um estudo utilizou amostra de canto. Em alguns estudos, a intensidade de amplificação da voz foi controlada, e em outros estudos essa variável não foi controlada. As variáveis de desfecho mais estudadas foram a análise acústica, com foco na modificação da pressão sonora e a autopercepção dos sujeitos. Os estudos foram concordantes ao observar que há redução no nível de pressão sonora quando o feedback auditivo é amplificado e que há efeitos positivos na produção da voz durante a amplificação nas amostras estudadas.

**Quadro 1:** Estudos sobre as manipulações de intensidade do feedback auditivo (Efeito Lombard, Efeito Sidetone e efeito Sidetone/Lombard) conforme autoria, ano de publicação, país do estudo, objetivo, método e conclusões

***Efeito Lombard***					
**Autor, país, ano**	**Objetivos**	**Amostra**	**Tarefa**	**Variáveis**	**Principais achados e conclusão**
1. Alghamdi et al., Inglaterra, 2018	Caracterizar as modificações acústica, fonética e articulatória da fala no efeito Lombard	54 palestrantes, ambos os sexos.	Ler 100 frases aleatorizadas Com exposição ao ruído de 80dB.	f0, média de volume, energia espectral, média da duração da vogal	Houveram modificações acústicas e articulatórias para todos os participantes. No maior aumento na duração estimada da vogal houve uma redução expressiva na frequência do segundo formante.
2. Castro et al., Chile, 2018.	Comparar os parâmetros acústicos aerodinâmicos, biomecânicos e neurofisiológicos de indivíduos saudáveis e com disfonia na exposição do efeito Lombard.	10 indivíduos, ambos os sexos, saudáveis e com disfonia por tensão muscular.	Pronunciar uma série de vogais e sílabas, exibidas em uma tela, sem e com ruído de 80dB e após o uso do ruído.	Videolaringoscopia, medidas aerodinâmicas e acústicas.	Sujeitos com disfonia por tensão muscular podem ser mais sensíveis ao efeito Lombard e terem maior dificuldade para retornar ao ajuste usual. Acredita-se que esses pacientes tem uma integração do controle auditivo-motor interrompida durante a produção da fala.
3. Fernandes et al., Brasil, 2018	Avaliar a influência do *feedback* auditivo na intensidade e na frequência da voz, em indivíduos sem queixas vocais	40 mulheres sem queixas vocais.	Produzir a vogal /a/, dizer os dias da semana e cantar antes, durante e após exposição a 80 dB de ruído branco por fones de ouvido.	Intensidade e frequência da voz, antes, durante e após a exposição ao ruído.	A condição de exposição ao ruído provoca aumento da intensidade da voz e a interrupção da exposição ao ruído ocasiona diminuição da intensidade vocal em mulheres sem queixas.
4. Lijima et al. Japão, 2016.	Investigar os efeitos do mascaramento do *feedback* auditivo em tarefa de canto.	6 homens	Cantar uma vogal /a/ firme nos tons: C3, G3 e C4 por 5 segundos sob ruído rosa de 85 dB, ruído rosa de 85 dB com filtro passa baixa de 2 kHz e sem mascaramento.	F0, nível de pressão sonora e formantes 1 e 2 em cada condição.	O nível de pressão do som e das frequências do formante 1 e 2 aumentaram sob o ruído em ambos os experimentos. O nível de pressão sonora e formante 1e 2 frequências diminuíram quando o filtro passa-alta de 2 kHz foi usado.
5. Kleber et al., Canadá, 2016.	Testar como o mascaramento do *feedback* auditivo afeta a precisão de afinação e atividade cerebral correspondente em cantores treinados e não treinados.	22 cantores, (4 M e 19 F), alocados em treinados e não treinados.	Ouvir e cantar os tons (Entre C# 3 a D5 para mulheres e F2 e B3 para homens), com e sem mascaramento inserido por fones de ouvido.	Ressonância magnética durante a exposição ao ruído.	A correspondência de precisão do *pitch* não foi afetada pelo mascaramento em cantores treinados, mas diminuiu em não cantores. A ínsula direita foi regulada para cima durante o mascaramento em cantores, mas regulada para baixo em não cantores. A conectividade funcional com parietal inferior, frontal e as áreas sensório-motoras relevantes para a voz aumentaram nos cantores, mas diminuíram em não cantores.
6. Yiu e Yip. China, 2015.	Investigar os efeitos do ruído ambiental na intensidade vocal e f0 usando um acelerômetro.	24 jovens adultos (2 grupos de 12 F e 12 H).	Ler texto, de 3 a 5 minutos, nas condições:1. sala silenciosa (35,5 dB);2. sala com nível moderado de ruído ambiente (54,5 dB); 3. uma sala com ruído ambiente alto (67,5 dBA).	variações de F0, percepção de esforço, nível de pressão sonora e medidas de dose vocal.	Ambos os grupos apresentaram aumentos na intensidade vocal, F0 e percepção do esforço vocal no ambiente de alto ruído do que nas outras duas condições. Os resultados apoiam que os níveis de ruído para conversação devem ser mantidos <50–55 dB para manter a inteligibilidade da fala.
7. Erdemir e Rieser. EUA, 2012.	Investigar os efeitos da exposição ao ruído no controle das habilidades de canto em cantores treinados e não treinados.	42 indivíduos; ambos os sexos. Grupo cantor, grupo instrumentistas e grupo de não músicos.	Cantar uma mesma música sob as condições com e sem mascaramento (som de conversação e música) inserindo por fones de ouvido, em 95dB. Os participantes foram instruídos a tentar manter a intensidade vocal em 80 dB a partir de *feedback* visual.	**Análise** acústica, variações da F0.	O *feedback* auditivo é um fator importante para manter a precisão do tom e do tempo mesmo após anos de treinamento musical. Cantores dependeram menos do *feedback* auditivo. Os instrumentistas e não músicos foram prejudicados pela ausência do *feedback* auditivo.
8. Li e Jeng. China, 2011.	Examinar a manutenção do tom da voz, durante modificações no *feedback* auditivo observando os efeitos da relação sinal ruído em várias intensidades de estímulo.	12 adultos (5 M e 7 M)	Produzir vogal /i/ sob seis condições de relação sinal ruído (12, 6, 0, 6 e 12 dB) em três diferentes intensidades de mascaramento auditivo por fones de ouvido (70, 55 e 40 dB) durante a fonação.	Eletroencefalografia, variações da F0.	Existe uma tolerância de controle do *pitch* em relação ao ruído. Existe uma relação sinal ruído mínima para avaliar processamento do *pitch.*
9. Caldeira et al., Brasil, 2011.	Verificar e comparar a ocorrência das modificações vocais de repórteres e não-repórteres na presença de ruído mascarante.	46 sujeitos, ambos os sexos alocados: 23 repórteres e 23 não-repórteres.	Ler um trecho de uma matéria de jornal (36 palavras) sob as condições: sem ruído, com ruído de 50 dB e com ruído de 90 dB. Os ruídos foram inseridos por fones de ouvido	Avaliação perceptiva auditiva e análise acústica.	Com 50 dB de mascaramento, houve maior aumento nos parâmetros *pitch* (82,6%), *loudness* (91,3%) e tensão (82,6%) no grupo controle, quando comparado ao grupo dos repórteres. O mesmo ocorreu com ruído de 90 dB para os parâmetros *pitch* (95,7%), *loudness* (100%) e tensão (91,3%). Os repórteres demonstram conseguir inibir parcialmente o impacto negativo das situações de ruído.
10. Grillo et al., EUA, 2010.	Explorar os efeitos de mascaramento auditivo na resistência laríngea quando o indivíduo produzia uma voz soprosa, normal e tensa de mulheres treinadas.	18 mulheres vocalmente treinadas.	Produzir vozes soprosa, normal e tensa em 7 frequências fundamentais (220 Hz, 277 Hz, 349 Hz, 440 Hz, 554 Hz, 698 Hz e 880 Hz) durante uma emissão repetida de /pi/ sob *feedback* auditivo normal e mascarado com ruído de 104 dB.	Médias e desvio padrão da resistência laríngea, medidas aerodinâmicas.	Os valores da resistência laríngea para voz soprosa e normal permaneceram constantes em ambos os *feedback*s enquanto que para voz tensa, aumentaram no *feedback* mascarado. A voz tensa pode ser mais suscetível à influência do *feedback* auditivo porque é menos estável do que os outros padrões testados.
11. Lindstrom et al., Suécia, 2009.	Investigar as correlações entre o nível de ruído e a F0 em uma população de professores de pré-escola em seu local de trabalho.	13 professoras de pré-escola.	Atividade usual de docência de 3 à 4 horas de aula enquanto era mensurado a intensidade produção vocal (por meio de microfone acoplado próximo da boca, e o nível de ruído do ambiente (mensurado por decibelímetro).	Dose vocal, nível de pressão sonora da voz e o nível do ruído local.	O comportamento vocal em relação à exposição ao ruído é altamente individual com base nos parâmetros analisados. A redução do nível de ruído não acompanhou, necessariamente, a redução do nível de pressão sonora emitido pelo participante.
12 Larson et al., EUA, 2008.	Testar as modificações vocais na F0 e controle de amplitude durante perturbações simultâneas de tom de voz e *feedback* auditivo de intensidade.	24 sujeitos testados (2 M e 22 F).	Sustentar uma vogal /u/ nas condições: 1. Alterar a frequência em 0,5 semitons para baixo ou para cima; 2. Alterar a intensidade em 10 dB acima do sinal produzido; 3. Alterar a intensidade e a frequência nos padrões anteriormente apresentados.	Modificações de intensidade e F0.	Os sujeitos responderam à direção oposta dos estímulos de deslocamento de frequência ou intensidade. Dependendo da direção do estímulo, ambas as respostas podem mudar na mesma direção ou na direção oposta um do outro.
13. Lee et al., EUA, 2007.	Investigar a relação entre a função auditiva e a F0 usando mascaramento binaural com ruído durante vocalizações de vogais sustentadas.	8 indivíduos saudáveis (4 M e 4 F).	​​Produzir vogal sustentada /a/ nas intensidades de 65 a 75 dBA e 90 a 100 dBA com e sem a presença de 85 dB de ruído inserida por fones de ouvido.	Modificações da F0.	Houve um aumento na faixa de frequência de <3 Hz durante a inserção do ruído. Um controle de *feedback* negativo na F0 é sugerido em relação às modulações F0 menores que 3 Hz. O sistema auditivo ajuda a controlar a estabilidade da F0 durante a produção sustentada de vogais.
14. Ferrand, EUA, 2005.	Investigar a estabilidade fonatória medindo mudanças na intensidade, F0, jitter e NHR, em diferentes condições de ruído.	22 mulheres sem queixas.	Três emissões prolongadas da vogal /a/ para cada proposições: 1. nível de ruído (0-dB ML); 2. 50-dB de ruído inserido por fones de ouvido; 3. 80 dB de ruído inserido por fones de ouvido.	Medidas acústicas de frequência e intensidade.	Houve aumento na intensidade da produção vocal nas duas condições de ruído. Houve também um aumento na frequência fundamental (F0), porém, de forma menos robusta.
15. Deliyski et al., EUA, 2005.	Investigar a influência do ruído na precisão, confiabilidade e validade das medidas acústicas de qualidade de voz para gênero, idade, variabilidade intersujeitos e intrassujeitos.	20 participantes de ambos os sexos.	Produzir vogal sustentada /a/ por 10 segundos à 88dB, inseridos por fones de ouvido, durante a presença e ausência de ruído em relações de 42 dB acima, 30 dB acima e 30 dB abaixo da intensidade de produção.	Medidas acústicas,	Os resultados sugerem que o recomendado, aceitável e níveis inaceitáveis ​​de ruído no ambiente acústico estão acima de 42 dB, acima de 30 dB e abaixo de 30 dB de relação sinal-ruído, respectivamente.
16. Mürbe et al., Alemaha, 2003.	Avaliar o efeito do treinamento no controle do canto em tarefas de legato e staccato, andamento lento e rápido em alunos com 3 anos de educação musical.	22 cantores treinados, ambos os sexos.	Cantar a vogal /a/ em uma escala ascendente e um padrão de tríade descendente cobrindo toda a sua faixa de afinação, com e sem mascaramento de 105dB, inserido por fone de ouvido, em legato e staccato e em um ritmo lento e rápido.	F0 e comparação entre intervalos e afinação.	O mascaramento comprometeu a precisão da afinação, por staccato e legato, e por performance rápida em oposição à performance lenta. O *feedback* cinestésico contribui para a precisão da afinação em cantores treinados.
17. Mürbe et al., Alemanha, 2002.	Estimar a importância dos *feedback*s auditivo e cinestésico para o controle da afinação da voz em 28 alunos iniciantes profissionais de canto solo.	28 cantores, ambos os sexos, (17 F e 11 H).	Cantar a vogal /a/ em uma escala ascendente e um padrão de tríade descendente cobrindo toda a sua faixa de afinação, com e sem mascaramento de 105dB, inserido por fone de ouvido, em legato e staccato e em um ritmo lento e rápido	Medida da precisão da F0 por meio de Software.	O mascaramento comprometeu a precisão do *pitch* em 14% em todos os sujeitos nas condições do andamento rápido, staccato e legato. O *feedback* auditivo contribui para controle da afinação dos cantores.
18. Tonkinson. EUA, 1994.	Comparar o nível de resposta de intensidade vocal de adultos cantores com diferentes tempos de treinamento antes e após instruções verbais para resistir ao efeito *Lombard* ao cantar com uma fita pré-gravada de um coral cantando.	27 indivíduos de ambos sexos.	Cantar e ignorar, por meio de comandos verbais, o efeito L*o*mbard que era produzido a partir da inserção, por fones na intensidade de 80 a 100 dB, de áudio contendo gravação de outras pessoas cantando o mesmo trecho da música.	Nível de pressão sonora.	Ambos os grupos conseguiram resistir ao efeito *Lombard* a partir de ordens simples dadas pelo avaliador.
19. Herbert et al., EUA, 1988.	Testar a resistência do efeito Lombard quando os indivíduos são instruídos e treinados com *feedback* visual para suprimi-lo.	24 estudantes, ambos os sexos, alocados: G1 (intensidade vocal constante durante 2 minutos alternando períodos de silêncio e ruído com *feedback* visual para resistir ao efeito *Lombard*), G2 (As mesmas instruções que o G 1, mas sem *feedback*), G3 (sem instrução nem *feedback* visual).	Falar espontaneamente durante a sessão por 20 minutos enquanto recebiam o ruído branco de 90 dB por fones de ouvido.	Variações da intensidade de fala.	Os indivíduos que tinham o apoio visual, conseguiram inibir a resposta de *Lombard* e a inibição permaneceu depois que o *feedback* visual foi removido. A resposta do *Lombard* é, em grande parte, automática e involuntária.
***Efeito Sidetone***					
**Autor, país, ano**	**Objetivos**	**Amostra**	**Tarefa**	**Variáveis**	**Principais achados e conclusão**
1. Nudelman et al., EUA, 2021,	Examinar os efeitos da amplificação do feedback auditivo por condução óssea nos parâmetros vocais acústicos e na autoavaliação subjetiva do esforço vocal em pacientes com distúrbios vocais.	47 disfônicos (14H e 13M)	Realizar leitura de textos sob três condições: amplificação do feedback auditivo em 54 dB NA e 58 dB NA, e sem amplificação. Amplificação realizada por microfone e fones de ouvido	Esforço vocal autopercebido e registrado em EVA, Nível de adaptação da pressão sonora da voz	Existe uma adaptação positiva consistente no nível de pressão sonora hiperfunção vocal, insuficiência glótica e patologias laríngeas quando submetidos a tarefa de amplificação com maior intensidade (58dB).
2. Tomassi et al., EUA, 2021	Determinar o potencial terapêutico dos efeitos da amplificação na função vocal durante a telecomunicação audiovisual	18 participantes (8H e 10M).	Tarefa de conversação em três condições: sem amplificação, com amplificação *sidetone* baixa e elevada, durante 10 minutos	Intensidade vocal, qualidade vocal e esforço autopercebido.	Houve diminuições na intensidade vocal durante a condição de amplificação do *feedback* auditivo e os participantes perceberam menos esforço vocal durante a amplificação. Resultados indicaram possível melhora da qualidade vocal
3. Assad et al., Brasil, 2017	Determinar se a amplificação da voz influencia na dose vocal em professoras com disfonia.	15 professoras com disfonia funcional.	Dois momentos de avaliação.1º Momento: lecionar com sistema eletrônico portátil de amplificação sonora por 92 minutos; 2º Momento: lecionar sem sistema de amplificação sonora por 92 minutos.	Intensidade, frequência fundamental, porcentagem de fonação, dose cíclica e dose distância.	O uso de amplificação vocal em professores promove uma redução da F0 e da intensidade da voz. As doses de ciclo e doses de distância mostraram que a amplificação permite que o professor mantenha o mesmo tempo de fonação, mas diminui o número de oscilações das pregas vocais (dose cíclica) e a distância total percorrida pelo tecido das pregas vocais durante a fonação (dose à distância), reduzindo a exposição das pregas vocais ao trauma vocal.
4. Gaskill et al., EUA, 2011	Determinar o efeito de um amplificador de voz portátil na dose vocal em professores com e sem queixas vocais.	2 professoras, com e sem queixas vocais.	Lecionar uma semana com e uma semana sem uso de um sistema de amplificação da voz portátil e com uso de dosímetro vocal todo o experimento. Cada semana durou cinco dias contando um turno por dia.	Cíclica, dose de distância e intensidade.	O uso do amplificador foi eficiente para reduzir a carga vocal, devido a diminuição da intensidade de fala. A amplificação reduz a dose de distância e parece diminuir a dose cíclica.
5. Nsdottira et al., Islandia, 2003.	Investigar as mudanças na qualidade da voz dos professores durante duas situações: uma jornada de trabalho em condições normais e outra com amplificação de som.	5 professores (3M e 2H)	Lecionar durante o dia de trabalho mais difícil em condições normais e amplificadas por sistema de microfone e caixa de som calibrada para sinal máximo de 80 dB. As gravações foram realizadas na primeira e última aula do turno do professor com duração de 40 minutos cada.	F0, nível de pressão sonora e questionários contendo a opinião dos participantes e análise acústica.	Os professores relataram menos cansaço quando utilizaram o amplificador. As vozes gravadas, durante o uso do amplificador, foram consideradas menos tensas. Na análise acústica foi encontrada a diminuição da inclinação espectral nas vozes que utilizaram o amplificador.
6. McCormick et al., EUA, 2002	Examinar a eficácia do Sistema de Amplificação de Voz Portátil ChatterVox (Siemens Hearing Instruments) para reduzir o nível de pressão sonora (NPS) da voz de palestrantes durante uma aula simulada em sala de aula.	10 palestrantes.	Ler texto foneticamente balanceado, utilizando um amplificador de voz, acoplado a caixas de retorno do som. O participante tinha o *feedback* auditivo amplificado por 2 minutos de leitura e no meio da tarefa de amplificação era desligada e o participante lia por mais 2 minutos. O nível de adaptação da voz era medido próximo da boca e no fundo da sala.	Medidas de intensidade de voz.	Observou-se diminuição média na intensidade vocal em nível da boca de 6,03 dB NPS e um aumento médio de 2,55 dB NPS no fundo da sala. O dispositivo de amplificação ChatterVox reduz o nível de intensidade vocal no microfone.
7. Jónsdottir et al., Finlandia, 2002.	Investigar as mudanças na fala durante um dia de trabalho do professor em condições normais e ao usar um aparelho de amplificação da voz.	3 professoras e 2 professores	Lecionar durante um dia de trabalho intenso em condições normais e amplificadas por sistema de microfone e caixa de som calibrada para sinal máximo de 80 dB. As gravações foram realizadas na primeira e última aula do turno do professor com duração de 40 minutos cada.	F0, nível de pressão sonora e questionários contendo a opinião dos participantes.	Um aumento em F0 e nível de pressão sonora foi encontrado durante o experimento, mas a mudança foi maior na F0 quando a amplificação foi usada. Todos os professores relataram menos cansaço vocal ao usar a amplificação. Os resultados apoiam a sugestão de que um o aumento da F0 e nível de pressão sonora não são apenas um sinal de fadiga vocal, mas pode até refletir uma adaptação adequada à demanda vocal.
8. Jónsdóttir, Islandia, 2002.	Verificar se o uso de amplificação de som em sala de aula tem efeitos benéficos na produção e resistência vocal de professores Determinar os efeitos negativos da amplificação para o falante e o ouvinte.	33 professores e 791 alunos.	Professores lecionaram com e sem amplificação da própria voz durante uma semana em cada condição. O sistema de amplificação foi por meio de microfones de lapela e a recepção por meio de caixas de som externas.	Percepção dos alunos e autopercepção dos professores	97% dos professores relataram produção de voz mais fácil, 82% melhoraram a resistência vocal. 84% dos alunos acharam mais fácil ouvir e 63% dos alunos melhoram a concentração quando a amplificação foi usada. Os pontos negativos relatados por professores e alunos foram problemas técnicos com os aparelhos.
9. Laukkanen et al., Finlandia, 2002	Investigar os efeitos do HearFones pela *autopercepção*, analise perceptiva auditiva e acústica sobre a intensidade recebida, qualidade da voz nas tarefas de canto, vocalização e leitura	Teste 1: 2F e 2M. Teste 2: 9F, 4 M. Teste 3: 6 fonoaudiólogas.	Testes: 1 - Amostra de leitura de texto com e sem *HearFones*. 2 – Amostra de canto com e sem *Hearfones*. 3 – Emissão da vogal /pá/, leitura de texto e canto, com e sem *HearFones*.	**A**nalise acústica, eletroglotografia, analise perceptiva auditiva, autopercepção da qualidade e conforto vocal	O *HearFones* parece melhorar os harmônicos da voz e diminuir a intensidade vocal. Os participantes perceberam suas vozes “menos tensas” e “com melhor controle”. A eletroglotografia indicou melhor fechamento glótico e/ou diminuição da atividade do músculo tireoaritenóideo durante o uso do aparelho.
10. Roy et al., EUA, 2002	Comparar os efeitos das orientações sobre higiene vocal versus amplificação de voz em professores com problemas de voz.	44 professores com queixas vocais, alocados em 3 grupos: Grupo controle, grupo higiene vocal e grupo amplificador portátil sonoro (*Chatter Vox*).	Os participantes de cada grupo foram instruídos a utilizarem as estratégias de higiene vocal, uso do amplificador ou nenhuma intervenção de acordo com sua alocação no grupo por seis semanas	Autopercepção de desvantagem vocal e gravidade do problema de voz e percepção da estratégia utilizada, análise acústica e perceptiva auditiva.	Não houve diferenças entre os grupos de amplificação sonora e higiene vocal. O grupo da amplificação relatou maior clareza e maior facilidade de produção de voz com maior adesão à estratégia proposta. As descobertas apoiam a utilidade clínica da amplificação sonora como alternativa para na reabilitação dos problemas vocais em professores.
11. Nsdottir et al., Finlandia, 2000	Testar se a amplificação sonora reduz a carga de produção vocal	5 mulheres.	Leitura de texto com 133 palavras em circunstâncias normais, ouvindo sua própria voz amplificada, por fones de ouvido e com *feedback* auditivo amortecido por tampões de espuma inseridos no canal auditivo externo.	Modificações acústicas de F0 e nível de pressão sonora.	A F0, nível de pressão sonora e o primeiro formante diminuíram durante o *feedback* amplificado e amortecido. Os resultados sugerem que tanto a amplificação quanto o amortecimento do *feedback* auditivo podem reduzir a carga vocal durante a fonação.
12. Chang-Yit et al., EUA, 1975	Avaliar o efeito e a estabilidade da amplificação da própria voz em diferentes condições de amplificação ao longo do tempo.	Experimento 1 = 9 universitários; experimento 2 = 6 universitários.	Experimento 1: fala espontânea durante 12 min enquanto a voz era amplificada em 20dB; experimento 2: fala espontânea durante 6 min enquanto a voz era amplificada em 20dB ou 10dB e fala durante 6 minutos enquanto a voz era amplificada e adicionado um ruído 80 db. O experimento 2 foi repetido por 5 dias.	Modificações no nível de pressão sonora na modificação da voz.	O ajuste compensatório na voz no experimento 1 foi a redução de 7 dB para 20 dB de amplificação. No experimento 2 o efeito foi o mesmo em todas as repetições do experimento. A apresentação contínua do ruído não dessensibiliza o sujeito quanto ao efeito da inserção do ruído. O efeito de amplificação é um componente de regulação da fala.
***Efeitos Sidetone e Efeito Lombard***					
**Autor, país, ano**	**Objetivos**	**Amostra**	**Tarefa**	**Variáveis**	**Principais achados e conclusão**
1. Bottalico et al., EUA, 2016	Avaliar os efeitos na imprecisão da afinação entre as notas de referência e a nota cantada nas condições: 1) nível de *feedback* externo, (2) andamento (lento ou rápido), (3) articulação (legato ou staccato), (4) tessitura (baixa, média ou alta) e (5) direção de semi-frase (ascendente ou descendente)	20 sujeitos, ambos os sexos, alocados em cantores profissionais e semiprofissionais.	Cantar repetições de arpejos em diferentes andamentos e articulações sob as condições de *feedback* inalterado, *feedback* aumentado por painéis reflexivos e *feedback* diminuído por tampões de ouvido.	Precisão de afinação.	A imprecisão foi maior quando o andamento era mais rápido e a articulação do tipo staccato em cantores semi profissionais. Porém, cantores profissionais foram mais precisos na condição do *feedback* diminuído do que nas outras condições de *feedback* externo. Com o aumento do treinamento, a imprecisão da afinação do cantor diminui.
2. Bottalico et al., EUA, 2015	Analisar o efeito *Lombard*, A relação do nível de pressão sonora e do *feedback* auditivo, a relação entre a qualidade da voz e o *feedback* auditivo externo, nível de acompanhamento, registro de voz e o gênero do cantor em cantores profissionais e não profissionais.	10 cantores amadores e 10 cantores profissionais de ambos sexos.	Cantar trechos de uma mesma música nas condições: *feedback* auditivo sem alterações, amplificação e diminuição do *feedback* auditivo durante o uso de um acompanhamento musical em três níveis (70, 80 e 90 dBA) inserido por fones de ouvido.	F0, qualidade de voz.	O efeito *Lombard* foi mais forte para amadores, os níveis mais altos de *feedback* auditivo externo foram associados a uma redução no nível de pressão sonora e este efeito foi mais forte em cantores amadores. A melhor qualidade de voz foi detectada na presença de níveis mais elevados de *feedback* auditivo externo.
3. Bottalico et al., EUA, 2015	Avaliar os efeitos do estilo de voz (suave, normal e forte), nível de ruído de fundo e *feedback* auditivo externo sobre esforço vocal e conforto vocal autorreferido, controle e fadiga vocal.	20 indivíduos sem queixas.	Ler um texto de forma suave, normal e forte com duração entre 1 e dois minutos, em uma sala semi-reverberante com e sem painéis que aumentavam o *Feedback* auditivo, e em condições de ruído de 40 dB e de 61 dB	**A**utopercepção de fadiga, conforto e controle vocal	Os participantes aumentaram seu nível de fadiga na presença de ruído e quando instruídos a falar em um estilo forte. Eles diminuíram a fadiga quando o *feedback* foi aumentado e ao falar em um estilo suave. Na autopercepção, houve uma preferência pelo estilo normal sem ruído.
4. Siegel e Pick, EUA, 1995	Verificar as mudanças no nível de pressão sonora quando há aumento ou diminuição do *feedback* auditivo, na presença ou ausência de ruído em condições normais ou instruídas à compensar mudanças.	20 indivíduos de ambos sexos.	Falar espontaneamente durante amplificação ou redução do *feedback* auditivo de sua voz em 20 dB. Primeira instrução: realizar compensações necessárias para os diferentes tipos de manipulação. Na segunda instrução: não alterar o nível de pressão sonora em vista das modificações.	Modificações no nível de pressão sonora.	O efeito de redução ou amplificação foi maior quando os sujeitos foram instruídos a compensar as modificações de alterações no volume. A presença do ruído aumentou as respostas de compensação dos sujeitos. A presença do ruído aumenta a resposta às manipulações do *feedback* auditivo.

Quatro artigos, correspondendo a 6,66% dos encontrados, estudaram os efeitos da manipulação da intensidade, incluindo o efeito Lombard e o efeito *Sidetone,* na mesma pesquisa, e são do tipo experimental transversal. Os estudos realizaram tarefas diferentes para verificar os efeitos da amplificação da voz. As populações foram cantores (n = 2) e indivíduos sem queixas vocais (n = 2), de ambos os sexos (n = 4). Foram utilizadas amostras de leitura (n = 1), fala (n = 1) e canto (n = 2). As manipulações da intensidade foram realizadas com diferentes recursos, variando entre amplificadores eletrônicos, placas de reflexão acústicas, sistemas de retorno com fone e amplificadores acústicos. Entre as variáveis, os estudos utilizaram a análise acústica e a autopercepção de conforto. Os autores observaram melhora da qualidade vocal e do controle da F0 na amplificação do Feedback auditivo e o aumento do esforço na presença de ruído.

Foram encontrados 25 estudos, 41,67% dos encontrados, que investigaram a manipulação da frequência do *feedback* auditivo *(Pitch-Shift),* todos do tipo experimental transversal. Os estudos tinham como objetivo, em linhas gerais, observar o reflexo de mudança de tom e suas aplicações nas amostras estudadas. A maioria das amostras foi composta por ambos os sexos (n = 14), por pessoas sem queixas vocais (n = 24) e não profissionais da voz (n = 19). As tarefas mais utilizadas foram a emissão de vogais sustentadas (n = 18) e tarefa de canto (n = 3). A variável mais estudada foi a variação na frequência fundamental (n = 19) para avaliar o efeito e alguns estudos utilizaram de outras variáveis para entender o reflexo, tais como a magnitude e direção da resposta reflexa, eletroencefalografia, eletroglotografia, imagens laríngeas, tempo de resposta, medidas cepstrais. Em todos os experimentos observa-se uma resposta reflexa à mudança de tom no feedback auditivo, porém, com características individuais em cada estudo (Quadro 2).

**Quadro 2:** Estudos sobre as manipulações de intensidade do feedback auditivo *(Pitch-shift-reflex)* conforme autoria, ano de publicação, país do estudo, objetivo, método e conclusões

Autor, país, ano	Objetivos	Amostra	Tarefa	Variáveis	Principais achados resultados e conclusão
1. Larson et al., EUA, 2021.	Examinar se a referência interna da voz é fixa ou variável, comparando a f0 com as respostas a alterações no *feedback* auditivo em duas condições apresentação.	33 participantes (26 F e 7 M)	Vocalizar a vogal /a/ nas condições: *feedback* auditivo alterado e introduzido durante a fonação; o *feedback* auditivo alterado, apresentado antes do início da vocalização e removido durante a vocalização. As modificações foram de 0,25, 1 e 2 semitons.	Tempo, magnitude e a direção de resposta ao estímulo.	Não houve diferenças na latência ou magnitude da resposta entre as condições de tempo, indicando que para uma tarefa de vocalização de vogal sustentada, o referente interno não é fixo.
2. Alem et al., Canadá, 2021.	Testar a hipótese: a duração das respostas adaptativas a mudança de *feedback* auditivo e a adaptação no tempo empregado em cada frequência emitida, depende da tarefa realizada, seja ela cantar, ler ou vocalizar.	30 participantes (16 F e 14 M).	Cantar “Parabéns pra você”, ler um parágrafo de Harry Potter e vocalizar /a/, /e/, /o/ com e sem e mudança de 1 semitom no *feedback* auditivo.	Análise de afinação e Modificações da F0.	Os comandos motores adaptativos usados por indivíduos, com audição normal, são maleáveis por meio de alterações no *feedback*, talvez mais ao ler em voz alta do que ao cantar ou vocalizar. Mas, esses efeitos são revelados por meio de mudanças sutis nas variações do tom de voz.
3. Kothare et al., EUA, 2020	Testar o quanto a resposta de adaptação se opõe a mudança de *feedback* auditivo e o quanto varia em função da direção da mudança de *feedback* aplicado nos formantes vocálicos.	18 participantes (10 F e 8 M).	Emitir palavras pré selecionadas enquanto a frequência do primeiro e segundo formantes (F1 e F2) são alteradas de até 50 Hz acima e abaixo.	Medidas acústicas.	A adaptação acontece dependendo da direção do deslocamento aplicado no espaço do formante da vogal, independente da magnitude do deslocamento.
4. Alexandra Schenck et al., EUA, 2020	Avaliar a relação entre o controle do *feedback* auditivo e a qualidade vocal medida pela proeminência do pico cepstral suavizado (CPPS), refletidos nos harmônicos do sinal de voz.	25 adultos saudáveis.	Produzir vogais sustentadas enquanto o *feedback* auditivo é modificado na intensidade (0, 3 ou 6 dB) e na frequência (0, 50 ou 1 semitons).	Proeminência do pico cepstral suavizado. (CPPS)	O aumento e diminuição de intensidade causaram aumento relativo no CPPS, indicando uma melhora nos harmônicos da voz, mesmo nos casos em que a intensidade vocal foi reduzida. Resultados indicam que há um mecanismo de controle da qualidade de voz que aumenta a harmonia do sinal de voz para melhorar a audibilidade na presença de variabilidade imprevisível na intensidade.
5. Behroozmand et al., Alemanha, 2020.	Investigar como a estimulação por corrente contínua transcraniana de alta definição do córtex motor ventral esquerdo modula os mecanismos neurais de integração sensório-motora durante o controle motor da voz.	30 participantes (20 F e 10 M).	Vocalizar vogal sustentada /a/ com variações de 1 semitom no *feedback* auditivo para cima e para baixo.	Estimulação por corrente contínua transcraniana de alta definição do córtex motor ventral esquerdo em duas correntes (1 mA ou 2 mA).	Não há um efeito diferencial de modulação de 1 mA versus 2 mA. A neuro estimulação do córtex motor ventral esquerdo modula os mecanismos sensório-motores controlem motor de voz subjacente.
6. Hilger et al., EUA, 2019.	Investigar como a direção e o tempo de uma perturbação no *feedback* auditivo do tom de voz, durante a produção de frases modula a magnitude e a latência do reflexo de mudança de tom.	32 participantes (21 F e 11 M).	Produzir três conjuntos de frases enquanto são aplicadas perturbações de pitch (2 semitons acima ou abaixo) no *feedback* auditivo por fones de ouvido na primeira, segunda ou terceira palavra de cada frase.	Acústica da voz e variações da f0.	O reflexo de mudança de tom foi maior após perturbações na primeira palavra da frase. A produção da palavra do final da frase foi acusticamente melhorada após perturbações no início da frase, mas, mais ainda depois perturbações na primeira palavra da frase. Os participantes podem integrar comandos corretivos de erros baseados em *feedback* revisando planos motores de alvos de entonação antecipada relativos a acústica na produção frasal.
7. Ziethe et al., Alemanha, 2018.	Analisar a funcionalidade dos mecanismos de controle da fonação e da fala entre pacientes com disfonia por tensão muscular (DTM) e indivíduos normais.	61 indivíduos saudáveis e 22 com DTM.	Fonação sustentada /a/ e fala com *feedback* auditivo alterado em 7 semitons para baixo ou para cima, inserido por fones de ouvido.	**E**letroencefalografia, eletroglotografia, sinal acústico da voz e sinal de vídeo.	Houve modificações em ambos os grupos entre a condição “sem *pitch*” e “*pitch*” das duas condições em relação à dinâmica das pregas vocais e à qualidade da voz. Pacientes com DTM mostraram mais irregularidades vibratórias durante a modificação do *feedback* do que os controles. Pacientes com DTM parecem ter uma interação perturbada entre os aspectos auditivo e cinestésico.
8. Alsius et al., EUA, 2017.	Testar se modificações do alvo de fala têm impacto no ajuste fino dos comandos motores vocais.	64 mulheres	Produzir a palavra “cabeça” enquanto o *feedback* auditivo recebido foi alterado por mudança sistemática nos primeiros formantes da vogal /e/ (de até 200 Hz em tempo real por fones de ouvido, enquanto era inserido avisos linguísticos e não linguísticos para correção.	Modificações da frequência fundamental.	Os comandos linguísticos induziram maiores comportamentos corretivos às perturbações acústicas do que os comandos não linguísticos. A correção automática de adaptações vocais é influenciada por mecanismos flexíveis e dependentes do contexto.
9. Arbeiter et al., Alemanha, 2017.	Investigar as alterações nos mecanismos de *feedback* auditivo e na qualidade da voz durante a fonação em resposta a uma mudança espontânea de *pitch* no *feedback* auditivo.	28 participantes.	Fonação da vogal /a/ ouvindo o *feedback* auditivo alterado em 7 semitons recebidos por fones de ouvido.	Eletroencefalografia (EEG), sinal de voz acústica, eletroglotografia(EGG), e videoendoscopia de alta velocidade (HSV).	O reflexo de mudança de tom foi detectado com sucesso em todas as variáveis utilizadas. Um aumento significativo das medidas de perturbação e um aumento dos valores dos parâmetros acústicos durante a mudança de tom foram observados, principalmente para o sinal de áudio. O mecanismo de *feedback* auditivo parece controlar não apenas o tom da voz, mas também a qualidade da voz.
10. Petermann et al., Alemanha, 2016.	Permitir a detecção do Reflexo de mudança de tom proporcionando análise detalhada do *feedback* cinestésico em trabalhos futuros.	5 participantes (2 H e 3 F).	Produzir a vogal /a/ e as sílabas /mama/ enquanto o *feedback* auditivo da voz era alterado em 7 semitons para baixo ou para cima inseridos em fones de ouvido.	Analise acústica da voz, eletroencefalografia, eletroglotografia e a imagens laríngeas.	O reflexo de mudança de tom foi encontrado em intervalos de latência fisiológica para EEG, EGG e sinais na acústica da voz. Também foi verificado com sucesso nos dados da dinâmica da laringe, obtido pelas imagens laríngeas, que mostraram sensibilidade semelhante como EGG e sinais de voz.
11. Behroozmand et al., EUA, 2015.	Investigar os mecanismos de controle motor do tom de voz, examinando a dinâmica espectro-temporal de sinais de EEG em não músicos (NM), músicos com ouvido relativo (RP) e músicos de ouvido absoluto (AP) durante mudança de tom.	34 sujeitos (11 não músicos, 12 músicos com ouvido relativo, 11 ouvido absoluto).	Manter vocalizações vogal /a/ enquanto recebem estímulos de mudança de tom de 1 semitom para baixo e para cima em seu *feedback* auditivo inserido por fones de ouvido.	Dinâmica espectro-temporal de sinais de EEG.	A ativação delta foi significativamente mais forte em NM. O teta evocado é um marcador neurofisiológico de aprimoramento do processamento de *pitch* em músicos e reflete mecanismos pelos quais os humanos incorporam o *feedback* auditivo para controlar o tom de sua voz. A ativação delta reflete os processos neurais adaptativos pelos quais os erros de produção vocal são monitorados e usados ​​para atualizar o estado das redes sensoriomotoras para a condução dos comportamentos vocais subsequentes.
12. Patel et al., EUA, 2015.	Investigar possíveis mecanismos automáticos que podem estar envolvidos no controle da frequência da voz nos limites de registro.	9 cantores (6 F e 3 H).	Cantar notas nas extremidades dos registros enquanto o tom do *feedback* auditivo da voz é inesperadamente alterado para o registro adjacente ou dentro do registro modal. As alterações foram inseridas por fones.	Modificações de F0 e eletroencefalografia.	Os cantores se adaptam a mudança súbita para o registro basal ativando mecanismos neurais que podem diminuir a magnitude de uma mudança na qualidade da voz.
13. Flagmeier et al., EUA, 2014.	Usar o modelo de equação estrutural e os dados de neuroimagem funcional para examinar propriedades neurais de uma voz com e sem modificação no *feedback* auditivo.	10 indivíduos (4 H e 6 M).	Vocalizar vogal /a/ durante 5 segundos intercalado com descansos, enquanto o *feedback* auditivo, inseridos por fones de ouvido, é alterado em 1 semitom para baixo ou para cima durante a fonação	Modelo de equação estrutural e os dados de neuroimagem funcional.	A presença de uma mudança de tom, que foi processada como um erro na vocalização, foi registrada como conexões alteradas entre giro temporal superior direito e esquerdo sendo que este último desempenha papel importante na detecção e correção de erros. Os resultados sugerem que o hemisfério direito é fundamental para a modulação do *pitch*.
14. Korzyukov et al., EUA, 2012.	Compreender a integração sensório-motora durante a vocalização, fala e seus componentes complexos	10 participantes (8 F e 2 H).	Vocalizar a vogal /a/ em tom habitual enquanto o *feedback* auditivo era alterado em 1 ou 4 semitons para cima e para baixo. Foram coletadas 100 amostras com e sem alteração de *feedback*.	Mudanças da F0, eletroencefalografia, modelagem causal dinâmica e potenciais relacionados à eventos.	Os resultados sugerem que tanto o giro temporal superior intrínseco, quantos conexões da esquerda para a direita são importantes na identificação de modificações de voz e na integração sensório motora. Modificações da própria voz e modificações que não são da própria voz são processadas ​​de forma diferente nos hemisférios direito e esquerdo.
15. Behroozmand et al., EUA, 2012.	Analisar as respostas vocais a perturbações do *pitch* no *feedback* da voz em que as tentativas de correção são classificadas de acordo com a direção da resposta e com a média em grupos de respostas ascendentes ou descendentes.	15 participantes (10 F e 5 M).	Vocalização da vogal /a/ no seu tom habitual de fala enquanto a frequência do *feedback* era alterada em 1, 2 ou 5 semitons para cima e para baixo em um total de 25 vocalizações.	Variações da F0.	A previsibilidade da direção e da magnitude do estímulo podem modular as respostas vocais às perturbações do tom de *feedback*.
16. Behroozmand et al., EUA, 2011.	Investigar os mecanismos neurais de controle de tom de voz para diferentes estímulos de modificação no *feedback* auditivo.	12 participantes (6 H e 6 F).	Vocalizações sustentadas da vogal /a/ enquanto *feedback* auditivo foi modificado em 2 semitons para cima. Os tipos de *feedback* eram: 1. Sua própria voz; 2. Um tom sinusoidal puro na frequência fundamental de sua própria voz; 3. tons com F0 e sua primeira frequência harmônica; 4. F0 com suas primeira e segunda frequências harmônicas; 5. F0 com seu primeiro, segundo e terceiro harmônico. Alterações inseridas por fones de ouvido.	Modificações acústicas, dados do eletroencefalograma.	Durante a produção vocal ativa, as amplitudes do reflexo de mudança de tom foram maiores em resposta às mudanças de tom na voz natural, moderadamente grande para estímulos complexos não vocais e menor para tons puros. Durante a escuta passiva, as respostas neurais foram igualmente grandes para mudanças de tom na voz e na comunicação não vocal com estímulos complexos, mas ainda maiores do que para tons puros.
17. Liu et al., China, 2011.	Investigar o envelhecimento das mudanças relacionadas no controle do *feedback* auditivo da voz durante a vocalização sustentada. Entender como as respostas vocais de F0 variam ao longo da vida adulta e com que idade as pessoas produzirão respostas vocais diferentes daqueles produzidos por jovens adultos.	60 indivíduos de ambos os sexos alocados em 5 grupos por faixa etária de idade.	Sustentar vogal /u/ enquanto o tom do *feedback* auditivo é alterado em 6,50 ou 61 semitons para cima e para baixo inseridos em fones de ouvido	Modificações da F0 e latências nas modificações do *pitch*.	As magnitudes de respostas aumentaram com o aumento da idade até que os valores máximos fossem alcançados para adultos de 51-60 anos de idade e então, diminuiu para adultos de 61-75 anos de idade. Adultos de 51 a 60 anos de idade também foram mais sensíveis à direção e magnitude das perturbações de *feedback* de pitch em comparação com adultos mais jovens. O reflexo de mudança de tom muda ao longo da vida adulta.
18. Larson et al., EUA, 2008.	Testar a hipótese que a eliminação da cinestesia estaria associada a uma maior resposta a uma perturbação externa da audição.	19 indivíduos (9 F e 10 H).	Vocalizar as vogais /u/ e /i/ com a modificação do *feedback* auditivo em 5 e 1 semitons para cima e para baixo nas condições com e sem anestesia das pregas vocais.	Modificações da frequência fundamental.	A anestesia das pregas vocais aumenta a resposta a uma perturbação auditiva imposta externamente. Existem diferenças para esses canais sensoriais: o *feedback* auditivo pode ser usado para o controle total da F0 enquanto a cinestesia é usada quando o *feedback* auditivo não está disponível.
19. Jones et al., Canadá, 2008.	Examinar as diferenças das modificações entre *feedback* de F0 e o sistema de produção vocal em cantores e não cantores.	40 participantes (20 cantores e 20 não cantores).	Cantar a silaba /ta/ enquanto *feedback* auditivo era deslocado para baixo em 1 Semitom.	Valores médios de F0.	Cantores confiam mais em modelos internos do que não cantores para regular as produções vocais. Os não cantores necessitam mais de pistas auditivas em tempo real.
20. Sivasankar et al., EUA, 2005.	Examinar se a F0 de um sujeito respondia não apenas a perturbações no tom de *feedback* de voz, mas também para mudanças com outros estímulos de tom apresentado congruente com o *feedback* de voz.	19 mulheres saudáveis.	Vocalizar a vogal /u/ em um tom habitual estável enquanto *feedback* era modificado em 1 semitom acima ou abaixo por fones de ouvido nas condições: 1. Retorno da própria de voz. 2. Retorno de tom puro. 3. Retorno da própria voz e tom puro.	Mudanças da F0.	Os sujeitos responderam à mudança da F0 ao invés de tons puro de *feedback* auditivo. O sistema áudio-vocal é sensível à mudança no tom de uma variedade de sons, o que pode representar um sistema flexível capaz de adaptar-se às mudanças na voz do sujeito. Esse sistema pode diminuir a influência de outros sons quando se tem o *feedback* da própria voz.
21. Leydon et al., EUA, 2003.	Demonstrar que as propriedades do sistema auditivo sustentam o vibrato iniciando respostas reflexas de mudança de tom enquanto os sujeitos produziram um tom contínuo.	6 participantes (5 F e 1 M).	Sustentar a vogal /E/ de forma estável enquanto o *feedback* auditivo é modulado em 0,25 semitons para baixo e para cima.	Mudanças e oscilações da f0 e funções de transferência da frequência.	As funções de transferência revelaram ganhos de pico em 4 a 7 Hz em todos sujeitos, com um ganho de pico médio em 5 Hz. Esses ganhos ocorreram na frequência nas regiões onde a saída de voz e os sinais de *feedback* auditivo estavam em fase. Um circuito de controle no sistema auditivo pode sustentar vibrato vocal e oscilações semelhantes a tremores na voz
22. Theresa et al., EUA, 2002.	Examinar se o componente inicial da resposta de mudança de tom é exclusivo para vocalizações de tom constante, ou se é um mecanismo que poderia ajudar no controle de vocalização de afinação estável e dinâmica.	30 cantores profissionais.	Vocalizações sustentadas da vogal /a/ e glissando enquanto o tom do *feedback* auditivo foi alterado para cima em 1 semitom e inserido com fones de ouvido.	Modificações da frequência fundamental.	As respostas de mudança de tom ocorreram durante vocalizações de glissando. Essas respostas apresentam latência maior e magnitude menor do que as respostas durante a fonação de nota estável. Essa resposta serve para trazer, automaticamente, o tom da fonação de acordo com um alvo pretendido, seja esse alvo constante ou não.
23. Liu et al., 2002.	Investigar o efeito do tempo de estímulo nas respostas vocais ao *feedback* com mudança de tom em padrões de entonação diferentes durante a produção da fala em mandarim.	10 participantes	Falar uma frase durante nas condições: 1. frequência fundamental (f0) da palavra final aumentada (entonação da pergunta); 2. frequência fundamental (f0) da palavra final ligeiramente diminuída (entonação da afirmação) ou modificação de 1 semitom no *feedback* apresentados em três momentos diferentes (160, 240 ou 340 ms) após o início da vocalização.	Modificações de f0 e da latência de resposta.	As magnitudes das respostas foram reduzidas para a condição de 340 ms em comparação com 160 ou 240 ms nas entonações. Uma mudança planejada na F0 pode causar uma modulação na resposta reflexiva. Há um período de tempo crítico durante o qual os mecanismos de resposta são mais sensíveis ao processo de planejamento.
24. Jones et al., Canadá, 2000.	Apresentar dados que abordam o papel desempenhado pelo *feedback* acústico no controle da F0 da voz.	18 homens.	Produzir vogal /a/ sob *feedback* com F0 normal, F 0 deslocado para cima e F 0 deslocado para baixo em 1 semitons	Modificações s na F0	Os sujeitos compensaram a mudança na F0. Quando o *feedback* de F0 voltou ao normal, os sujeitos modificaram sua F0 produzido de forma oposta direção para a mudança. Os resultados sugerem que a F0 é controlada por meio do *feedback* auditivo e com referência a um modelo interno de *pitch.*
25. Burnett et al., EUA, 1997.	Analisar as mudanças na F0 durante uma mudança de tom no *feedback* auditivo em um grupo de indivíduos normais e um pequeno número de cantores treinados.	67 participantes de ambos os sexos (15 cantores e 52 não cantores).	Emitir a vogal /a/ e cantar escalas ignorando as diferentes modulações frequência para baixo e para cima (0,25; 0,5; 1; 1,5; e 2 semitons) que eram apresentadas no *feedback* auditivo.	Modificações da f0.	96% dos indivíduos aumentaram sua F0 quando o tom de *feedback* foi diminuído, e 78% diminuíram sua F0 quando o *feedback* do *pitch* foi aumentado. Os resultados indicam que as pessoas confiam no *feedback* auditivo para controlar voz F0.

## DISCUSSÃO

Essa revisão de escopo mapeou a literatura disponível sobre as manipulações do *feedback* auditivo no controle motor da voz, em indivíduos adultos. Observa-se que há muitos artigos relacionados às diferentes manipulações. Porém, é possível observar que a maioria dos autores se detém a estudar a supressão *do feedback* auditivo (efeito Lombard*)* ^(23-37)^, e a manipulação do *pitch* por meio do efeito *pitch-shift-reflex* ^(5,38-60)^.

Destaca-se a necessidade de aprofundar os estudos do efeito da amplificação do *feedback* auditivo multidimensionalmente, sobre a voz. Cada estudo analisa um tipo de desfecho, há poucos elementos para se compreender as proporções entre amplificação de *feedback* auditivo e a diminuição da intensidade de voz.

Os estudos que pesquisaram o Efeito *Lombard* foram realizados com amostras distintas, como em adultos cantores, professores, indivíduos saudáveis, repórteres, indivíduos disfônicos e musicistas. Independente da população, a maioria dos resultados observados foi o aumento da intensidade de produção vocal, para as diferentes tarefas^(23-25,27,31,33,34,61,62)^. Poucos estudos observaram a supressão do *feedback* na população disfônica. Estudos mostram que essa população parece ser mais sensível aos efeitos da supressão do *feedback* auditivo e tem maior dificuldade de voltar à intensidade habitual de fala quando o ruído é retirado^(25,31,34,63)^.

No que diz respeito às variáveis de desfecho, grande parte das pesquisas, com efeito *Lombard* estudaram as modificações de intensidade e frequência da voz^(23,24,26-28,31-34,36,37,63,64)^. Algumas pesquisas incluem também a dose vocal^(27,31)^, avaliação perceptiva auditiva^(29)^, resistência laríngea^(30)^, medidas aerodinâmicas^(30,65)^, avaliação laríngea, duração de vogais, média de formantes^(10,24,65)^, ressonância magnética e eletroencefalografia^(25,28)^.

Não existe consenso quanto à intensidade do ruído mascarante para induzir o efeito *Lombard*, sendo que alguns pesquisadores utilizaram outros tipos de sinais, além de ruído por fones de ouvido, para mascarar a entrada auditiva, tais como música, ruído externo^(26,31,37)^. Os estudos observados utilizaram diferentes intensidades entre 40 a 100 dB sendo que, dentro dessa faixa, a intensidade de 90 dB é a que mais aparece nos métodos descritos^(29,33,63)^. Alguns estudos não estabeleceram intensidade fixa, baseando-se apenas no limiar individual dos participantes^(23,32)^. Isso mostra que ainda é necessário se pesquisar, minimamente, qual intensidade desencadeia o efeito *Lombard* e quais as diferenças entre as populações, uma vez que a literatura prescreve uma proporção observada entre o nível de ruído e o nível de amplificação da voz^(18)^.

Quanto aos principais achados do efeito *Lombard*, a maioria dos estudos afirma que a diminuição do *feedback* auditivo da própria voz provoca, inconscientemente, o aumento da intensidade da voz^(10,23-30,34,35,63,65)^, corroborando com a literatura a respeito do efeito^(11,12,18)^. Em linhas gerais, os autores encontraram aumento na dose vocal, aumento na intensidade da voz, imprecisão na correspondência de tom e maior susceptibilidade do efeito em vozes tensas^(25-27,30,31,36,63,65)^.

Além disso, os estudos observaram que o nível de modificação vocal é individual^(29,31)^ indicando, assim, a dificuldade de generalização e obtenção de uma resposta a respeito da proporção de intensidade do ruído e da produção de voz. Outro achado interessante é que os indivíduos podem controlar as modificações causadas pela supressão do *feedback* auditivo a partir de ordens simples ou com auxílio de *feedback* visual^(37,63)^.

Outra conclusão encontrada é que, o efeito *Lombard* empregado em cantores iniciantes, diminui a precisão da afinação em tarefas complexas, inferindo-se que os cantores iniciantes necessitam do auditivo para corresponder aos tons referenciados em tarefas complexas, bem como os cantores de nível avançado não dependem tanto do *feedback* auditivo para domínio de afinação já evidenciado na literatura^(25,27)^, assim como repórteres^(31,36,62,)^

Hipotetiza-se que o treinamento das habilidades cinestésicas auxilia no controle da intensidade da voz com menor influência do *feedback* auditivo para esse controle. Em contrapartida, os estudos com pacientes disfônicos mostraram que eles tendem a responder em maior magnitude ao efeito^(24,30)^

Quanto aos estudos de *feedback* do efeito *Pitch-Shift*, os pesquisadores têm incluído variáveis, tais como eletroencefalografia, dinâmica de espectro-temporal, neuroimagem funcional^(41,45-49,51)^ que verificam atividades corticais e associam atividades inter e intra hemisférios às mudanças reflexas de tom para entender profundamente a resposta a modificações de frequência no *feedback* auditivo^(41)^. Alguns estudos que analisaram neuroimagem e eletroencefalografia^(49)^ descreveram que o hemisfério direito desempenha papel fundamental na modulação do *pitch*.

Quanto à manipulação do *feedback*, os artigos descrevem modificações quando o *feedback* é alterado de 0,25 à 7 semitons para baixo ou para cima sendo que ainda não é definido o nível mínimo de manipulação para obtenção da resposta reflexa^(17,49-60)^. Em todos os experimentos é utilizado *hardwares* e *softwares* que modificam a frequência e fones de ouvido para apresentar o sinal modificado ao participante. Grande parte dos artigos selecionados com essa temática utilizaram modificações de um semitom em seus experimentos^(7,17,40,41,47,49,50,53-55,58,59)^.

As tarefas realizadas variam muito, sendo que há utilização de vogais sustentadas variadas em tom constante, corroborando com a literatura que descreve o *Pitch Shift-reflex* nessa tarefa^(10,15-17)^. Há experimentos que apresentam tarefas de trechos cantados^(38,48,54,59)^ ou emissão de silabas^(46,54)^, palavras e frases^(38,42,44,58)^, enquanto há o deslocamento da frequência no *feedback* auditivo. No que diz respeito às vogais sustentadas, não há padronização de qual vogal é utilizada, sendo que as mais recorrentes são as vogais /a/ e /u/^(17,38,41,43,45-47,49-56)^, porém, há artigos que utilizaram outras vogais^(38,45,51,54,56)^. As pesquisas observaram que há um reflexo de correção quanto há a manipulação da frequência do *feedback* auditivo, ou seja, se o *feedback* é alterado para cima, os participantes tendem a corrigir diminuindo a frequência de produção e vice-versa, assim como é descrito em outros artigos^(15-17,24)^.

A literatura disponível distingue resultados distintos quanto às populações vocalmente saudáveis e disfônicas, sendo que esta última parece apresentar resposta reflexa maior à mudança de tom no *feedback* auditivo^(42)^. No entanto, não foi encontrado ainda um padrão de forma quantitativa e proporcional da modificação frente à manipulação para indivíduos disfônicos e não disfônicos. Os estudos são concordantes ao observar que os cantores confiam mais em seu modelo interno de afinação do que pessoas não cantoras. Quanto maior o tempo de treino na tarefa de canto, o cantor tende a confiar mais em seu modelo interno de afinação do que no *feedback* auditivo, sendo que as manipulações não apresentam a mesma magnitude em cantores treinados se comparado a indivíduos não treinados^(47,48,54,57,59)^. Esses dados apontam a hipótese de que indivíduos treinados apresentam seu modelo interno bem estabelecido e pouco influenciado por modificações e atualizações externas em comparação aos indivíduos sem treino ou que apresentam algum tipo de alteração vocal.

Para testar o efeito *sidetone* foi utilizado fala espontânea e tarefa de leitura em diferentes populações estudadas, com maior ênfase em professores^(66-71)^ visto que esses são, constantemente, alvos do efeito *Lombard* da alta demanda vocal^(64,72,73)^. Das variáveis de desfecho, a mais utilizada foi a intensidade vocal, uma vez que era esperada a redução da intensidade no uso da amplificação^(61,63,69,72,74)^. Outras variáveis foram a autopercepção dos sujeitos e a análise perceptiva auditiva da voz^(60,68-71,74)^.

Dentre os sistemas para amplificar, os estudos não se concentram em uma forma específica, sendo que foi realizada amplificação por meio de amplificadores portáteis^(67,68,71,73)^ caixas de auto falante^(63,69,70,75)^ e fones de retorno^(60,74,76)^. Observando os achados, nota-se que todos os experimentos descreveram a redução do esforço e carga vocal em momentos que há a amplificação da própria voz, evidenciados no nível de pressão sonora no nível de esforço autopercebido^(60,61,63,66-71,74,75)^. Todos os estudos são conclusivos quanto à resposta de diminuição da intensidade de produção da voz, assim como já descrito na literatura^(60,61,63,66-71,74,75)^, e alguns descrevem ainda modificações na frequência da voz, concordando que há maior controle da voz quando há amplificação dela^(71,74,77)^.

Além disso, os estudos apontam resultados positivos na produção da voz, com menor esforço na fonação verificados na autopercepção^(68,71,74)^. Contudo, em cada experimento foi utilizado um o método é diferente e, embora a amplificação do *feedback* auditivo da voz, seja descrita como um recurso terapêutico que otimiza as práticas fonoterápicas, não se observou pesquisas que estudassem os seus efeitos, concomitantes à terapia da voz.

A utilização dos equipamentos também é divergente, sendo que não há comparações entre a efetividade das diferentes formas de se obter o mesmo tipo de manipulação. Evidenciou-se também poucos estudos que observaram as modificações e manipulações do *feedback* em indivíduos com disfonia, sendo necessários estudos de efetividade para verificar os resultados do uso da amplificação na reabilitação vocal^(66,67,71)^. Essas lacunas na literatura são campos de pesquisa que ainda necessitam de exploração científica.

Os estudos dessa revisão de escopo mostram que não há uma padronização de níveis de amplificação, nem há relação conclusiva entre o valor da diminuição da intensidade de produção vocal em resposta a quantidades específicas de amplificação do *feedback* auditivo. Poucos estudos exploram proporções ou níveis de intensidade durante os experimentos^(61,70)^. Essa lacuna dificulta o estabelecimento da relação ou correlação contrária da proporção observada no efeito *Lombard ^17^* .

Por outro lado, experimentos que associaram os efeitos da amplificação e a supressão do *feedback* auditivo tiveram também objetivos distintos, tais como avaliar precisão de tarefa em cantores, verificar níveis de pressão sonora em indivíduos normais e níveis de conforto^(61,77-79)^. Não se observou ao longo dessa revisão uma padronização de níveis de amplificação ou de inserção do ruído, a metodologia dos experimentos e as tarefas solicitadas (tarefas de canto, leitura de texto) são distintas entre eles.

A presente revisão de escopo buscou explorar os experimentos e condensar resultados das pesquisas com manipulação do *feedback* auditivo para o controle motor da voz de indivíduos adultos. Contudo, a diversidade metodológica dos experimentos, com informações às vezes escassas, tarefas de fala não padronizadas, variáveis de desfecho distintas e amostras reduzidas podem ter contribuído para uma limitação dos resultados. Por outro lado, a presente revisão apontou as lacunas do conhecimento existentes, estimulando a realização de pesquisas que abordem essa temática e, por consequência, contribuindo para um maior conhecimento acerca do treinamento ou terapias da voz.

## CONCLUSÃO

O mapeamento da literatura atual sobre os experimentos com o *feedback* auditivo, incluídos nessa revisão de escopo, evidencia que há diferentes métodos de promover a amplificação, supressão e manipulação da frequência do *feedback* auditivo. Os resultados, de forma geral, são similares quanto à resposta reflexa no controle motor da voz, observando as especificidades de cada experimento. No entanto, ainda é necessário uma melhor compreensão da relação entre a magnitude da manipulação e as respostas encontradas. Os principais achados das pesquisas acerca do *feedback* auditivo sobre o controle motor da voz evidenciam que, na supressão do feedback auditivo, o indivíduo tende a aumentar a intensidade da voz. Na amplificação do feedback auditivo, o indivíduo diminui a intensidade da voz e tem maior controle sobre a frequência fundamental e, nas manipulações da frequência, o indivíduo tende a corrigir a manipulação. Os poucos estudos encontrados que realizaram experimentos com indivíduos disfônicos mostram que eles se comportam de forma diferente dos não disfônicos.
